# Age-related gut microbiota succession in Neijiang pigs: insights for precision feeding and productivity

**DOI:** 10.3389/fmicb.2025.1698169

**Published:** 2025-11-12

**Authors:** Qihang Wu, Shijie Hu, Yan Wang, Yuanyuan Wu, Ye Zhao, Lili Niu, Xiaofeng Zhou, Linyuan Shen, Yihui Liu, Ying Chen, Mailin Gan, Li Zhu

**Affiliations:** 1 Farm Animal Germplasm Resources and Biotech Breeding Key Laboratory of Sichuan Province, Sichuan Agricultural University, Chengdu, China; 2 State Key Laboratory of Swine and Poultry Breeding Industry, Sichuan Agricultural University, Chengdu, China; 3 Key Laboratory of Livestock and Poultry Multi-omics, Ministry of Agriculture and Rural Affairs, College of Animal and Technology, Sichuan Agricultural University, Chengdu, China; 4 Department of Biological Engineering, Sichuan Fisheries School, Chengdu, China; 5 Sichuan Province General Station of Animal Husbandry, Chengdu, China

**Keywords:** Neijiang pig, 16S rRNA gene, metagenomic sequencing, gut microbiota, function capacity

## Abstract

**Objective:**

To characterize age-related gut microbiota succession in Neijiang pigs and translate these dynamics into actionable insights for precision feeding and productivity improvement.

**Methods:**

Growth data from 0 to 180 days (*n* = 16, 780 weight records) were fitted with three non-linear models to determine the optimal growth curve and partition physiological stages. Fresh feces were collected at 25, 70, 110, and 150 days (*n* = 6/stage). 16S rRNA V3–V4 amplicon sequencing was used to profile microbiota composition and diversity; PICRUSt2 was employed to predict metagenome functions against the KEGG database.

**Results:**

The Gompertz model best described growth (*R*^2^ = 0.996) with an inflection point at 84.2 days (25.9 kg). Microbial alpha-diversity (Shannon, Chao1) increased with age and plateaued after 110 days. Firmicutes and Bacteroidota dominated (>90% relative abundance), whereas Spirochaetota and Euryarchaeota expanded significantly in finishing pigs. LEfSe identified 45 stage-specific biomarkers: Prevotella_9, Collinsella and Blautia characterized suckling–weaning stages; Faecalibacterium and Clostridium_sensu_stricto_1 peaked at 70 days; Lactobacillus was dominant at 110 days; Treponema, Streptococcus and Bacteroides defined the 150-day microbiome. Functional prediction revealed a metabolic shift from basal biosynthesis and DNA repair in early life toward enhanced ABC transporters, bacterial motility proteins, oxidative phosphorylation and methane metabolism in finishing pigs.

**Conclusion:**

Our data provide a temporal blueprint of gut microbiota maturation that mirrors host nutrient requirements across growth phases. These microbial indicators and functional signatures can guide stage-specific dietary formulations and microbiota-targeted interventions to improve feed efficiency, reduce environmental emissions and enhance the productivity of indigenous pig breeds.

## 1 Introduction

The gut microbiota plays a crucial role in nutrient metabolism, immune modulation, and overall health in pigs (Isaacson and Kim, 2012; Wylensek et al., 2020; Hu et al., 2023). Its composition is dynamic and influenced by factors such as age, diet, environment, and host genetics (Ma et al., 2022; Zhao et al., 2019; Tan et al., 2018). Understanding the developmental trajectory of the gut microbiota across different growth stages is essential for optimizing feeding strategies and improving production efficiency, particularly in local pig breeds with unique genetic backgrounds.

The first comprehensive pig gut microbial gene catalog, released in 2016, was built from metagenomic sequencing of 287 fecal samples and encompasses 719 microbial species. The community is dominated by Firmicutes, Bacteroidetes, and Proteobacteria (Wang et al., 2019). A subsequent meta-analysis of 16S rRNA datasets from 20 pig microbiota surveys further designated *Prevotella*, *Clostridium*, *Alloprevotella*, *Ruminococcus*, *RC9*, *Blautia*, *Lactobacillus*, *Roseburia*, and *Subdoligranulum* as the core taxa consistently present in the porcine intestine (Holman et al., 2017). Despite these foundational studies, there remains limited research on the gut microbial composition and functional potential of Neijiang pigs, a traditional Chinese indigenous breed known for its adaptability, disease resistance, and meat quality (Zhenjian et al., 2023; Dan et al., 2024).

Comparative studies on gut microbiota dynamics across different pig breeds revealed that Jiaxing Black pigs (a local breed) exhibit higher microbial species richness and a greater number of unique bacterial communities in their fecal microbiomes compared to Duroc-Landrace-Yorkshire hybrid pigs (an imported breed) (Huang et al., 2024). The Duroc-Landrace-Yorkshire breed demonstrated superior digestive and nutrient absorption rates, while Jiaxing Black pigs exhibited stronger antioxidant stress resistance and greater tolerance to coarse feed. Taking Meishan pigs as an example, their intestines harbor abundant microorganisms such as Bacillus species, which produce multiple antimicrobial peptides (Wu et al., 2024). These peptides act like precision-guided missiles, targeting the growth and reproduction of common pathogens like *Escherichia coli* and Salmonella. This significantly enhances the Meishan pig’s resistance to intestinal diseases, allowing it to maintain good health even under relatively extensive farming conditions. These studies confirm significant differences in gut microbiota between local and imported pig breeds, providing compelling insights into the potential mechanisms underlying productivity variations among pig breeds.

To address these knowledge gap, we selected four key time points (25, 70, 110, and 150 days of age) spanning from birth to market weight (~180 days). To accurately model growth dynamics, we employed three commonly used mathematical models (Gompertz, Logistic, and Von Bertalanffy) (Chen et al., 2023) to fit growth curves from 0 to 180 days of age. This approach allowed us to identify the best-fitting model and determine the inflection point, which was used to divide the growth period into distinct stages for further analysis.

In addition, 16S rRNA sequencing was used to characterize the gut microbiota composition and diversity at each stage. Recent studies have highlighted the importance of primer selection and sequencing depth in accurately profiling microbial communities (Kameoka et al., 2021). We hypothesized that distinct microbial profiles and functional adaptations would be associated with each growth stage, reflecting the physiological and metabolic transitions of the host (Zhou et al., 2025). This study provides the first age-resolved investigation of the Neijiang pig—a slow-growing, coarse-fiber-tolerant breed renowned for its meat quality yet understudied in microbiome literature. Our findings provide new insights into the dynamic relationship between host development and gut microbiota, offering a basis for optimizing feeding practices and enhancing the production performance of local pig breeds.

## 2 Materials and methods

### 2.1 Ethics statement

All experimental procedures described below were approved by the Animal Ethical and Welfare Committee of Sichuan Agricultural University, Chengdu, China (Approval No. 2021302137, approval date: 1 July 2021).

### 2.2 Animals and samples preparation

In this study, all pigs were sourced from the same pig farm of a company in southwest China, including 20 Neijiang pigs (NN). The pig barn in Neijiang features fully slatted flooring and mechanical ventilation, with water curtain cooling in summer and geothermal heating in winter. Manure is removed once daily, followed by high-pressure washing between batches and a 3-day empty period. All pigs were raised in the same breeding environment, adopted the same feeding mode, and fed the same batch of the same feed. Diets were formulated to meet the Neijiang pig nutrient specifications (DB5110/T 77-2024): 3.0–3.2 Mcal DE kg^−1^, 14–16% CP, 0.75–0.85% SID lysine, 0.70% Ca and 0.60% P across grower-finisher phases. A corn-soybean meal basal diet, supplemented with 8% wheat bran and 3% rapeseed meal to match the breed’s higher crude-fiber tolerance, was fed ad libitum in meal form without in-feed antibiotics or zinc oxide throughout the tria. A total of 780 weight measurements were taken from birth to day 180 for the Neijiang pigs, which were used to fit growth curves. Fresh fecal samples were collected from individual pigs (NN, *n* = 6) at different growth stages (days 25, 70, 110, and 150). Each fecal sample was randomly collected from the herd, with the time from defecation to collection not exceeding 1 min to ensure sample freshness. At each growth stage, six different pigs were randomly selected from at least three litters and two pens (3 pigs/pen); no animal was sampled more than once. Thus, *n* = 6 represents independent biological replicates per time point, not longitudinal samples. Fecal samples were immediately immersed in liquid nitrogen upon collection and stored in 2 mL centrifuge tubes for transportation, then stored at −80 °C until DNA extraction.

### 2.3 Growth curve models

Growth curve fitting analysis was performed on Neijiang pigs using three commonly used growth curve models: Logistic (Shen et al., 2019), Gompertz (Winsor, 1932), and Von Bertalanffy (Malhado et al., 2009) (Table 1). Origin 2024 software was used to estimate model parameters A, B, K, m, and comparison indicators.

**Table 1:** Growth curve models adopted in the study and related parameters.

Model	Equation	Parameters	Wi , kg	Day at inflection	Maximum daily gain, g
Logistic	$W_{t}=A/(1+\text{Bexp}(-\mathrm{Kt}))$	A,B,K	A/2	(lnB)/K	KWi/2
Gompertz	$W_{t}=\text{Aexp}(-\text{Bexp}(-\mathrm{Kt}))$	A,B,K	A/e	(lnB)/K	KWi
Von Bertalanffy	$W_{t}=A{\left(1-B\exp(-\mathrm{Kt})\right)}^{3}$	A,B,K	8A/27	(ln3B)/K	3KWi/2

$W_{t}$ , body weight in kg at the time; t, age in days; A,B,K , specific parameters in the function; Wi , Weight of inflection; *e*, Euler number.

### 2.4 Pearson correlation and path analyses

To investigate the regularity of body conformation indices and body weight (BW) at different developmental stages, we measured the body conformation of 16 Neijiang pigs from birth to 180 days of age. These measurements were divided into early (before the inflection point age) and finishing (after the inflection point age) growth stages. Differences between the two growth stages were compared without considering genetic background. Body conformation indices included body length (BL), body height (BH), chest circumference (CC), abdominal circumference (AC), hip circumference (HC), chest width (CW), chest depth (CD), and cannon bone circumference (CBC). Pearson correlation and path analysis were performed using SPSS software (v.26.0; SPSS Inc., Chicago, IL, United States). Path analysis enables the study of direct and indirect effects simultaneously with multiple independent and dependent variables; thus, path analysis was used to partition the relative contributions of body size indexes using standardized partial-regression coefficients.

### 2.5 DNA isolation and 16S rRNA sequencing

Total genomic DNA was extracted using the CTAB/SDS method (Green et al., 2012). DNA concentration and purity were verified by gel electrophoresis in 1% agarose gel. An aliquot of the sample was placed into a centrifuge tube and diluted to 1 ng/μL with sterile water. PCR amplifications were performed using the diluted genomic DNA as a template. The V3-V4 hypervariable regions of the 16S rRNA were amplified (Caporaso et al., 2011). PCR amplification was performed using Phusion High-Fidelity PCR Master Mix (New England Biolabs, Ipswich, MA, United States) following the manufacturer’s instructions. PCR products were purified by agarose gel electrophoresis on 2% agarose gels. PCR products of between 400 and 450 bp were selected for PCR product purification using GeneJET (Thermo Scientific, Waltham, MA, United States) following the manufacturer’s instructions.

### 2.6 Data analysis

All sequencing data analysis was performed on Novogene’s cloud platform. First, paired-end reads were demultiplexed by unique barcodes and trimmed to remove barcodes and primer sequences. All steps were executed in Python 3.6.13; adaptors were removed through cutadapt (V3.3). Overlapping paired-end reads were merged into raw tags using FLASH v1.2.11.^1^ Raw tags were quality-filtered with fastp v0.23.1 to generate high-quality clean tags. Clean tags were screened for chimeras against the Silva (16S^2^) and UNITE (ITS^3^) reference databases, and chimeric sequences were removed with vsearch v2.16.0^4^ to yield the final effective tags: each sample retained 62,059–100,017 effective tags (median 79,220; mean 79,220 ± 11,634). OTU clustering (using a 97% similarity threshold) and species classification analysis were performed based on the clean data to determine the taxonomic information of microorganisms in the samples. OTU clustering was performed with the Uparse algorithm (Uparse v7.0.1001^5^). All Effective Tags from every sample were clustered at the default 97% identity threshold. For each OTU, the sequence with the highest frequency among all members was selected as the representative sequence for downstream taxonomic annotation. The abundance of OTUs was calculated, and alpha diversity analysis (including Chao1, Shannon index, etc.) was performed to assess the richness and evenness of species within the samples. Differences in community structure between different groups were analyzed using methods such as Principal Co-ordinates Analysis (PCoA), Principal Component Analysis (PCA), and Non-metric Multidimensional Scaling (NMDS). Additionally, use LEfSe analysis to identify significant differences in species (biomarkers) between different groups, and utilize PICRUSt2(V2.3.0) software to perform functional prediction analysis on the microbial communities in the samples. Functional profiling was performed with PICRUSt2 v2.3.0 using the default pipeline: OTUs were placed onto the IMG/M 5.0 whole-genome phylogeny, gene content was inferred with the castor hidden-state prediction algorithm, and KO abundances were mapped to MetaCyc v24.0 pathways via MinPath; KEGG pathway tables were also generated. No custom reference tree, weighting file, or additional parameters were applied. All analysis results are visualized through charts generated by the cloud platform.

## 3 Results

### 3.1 Comparison of growth curve models

**Figure 1:**
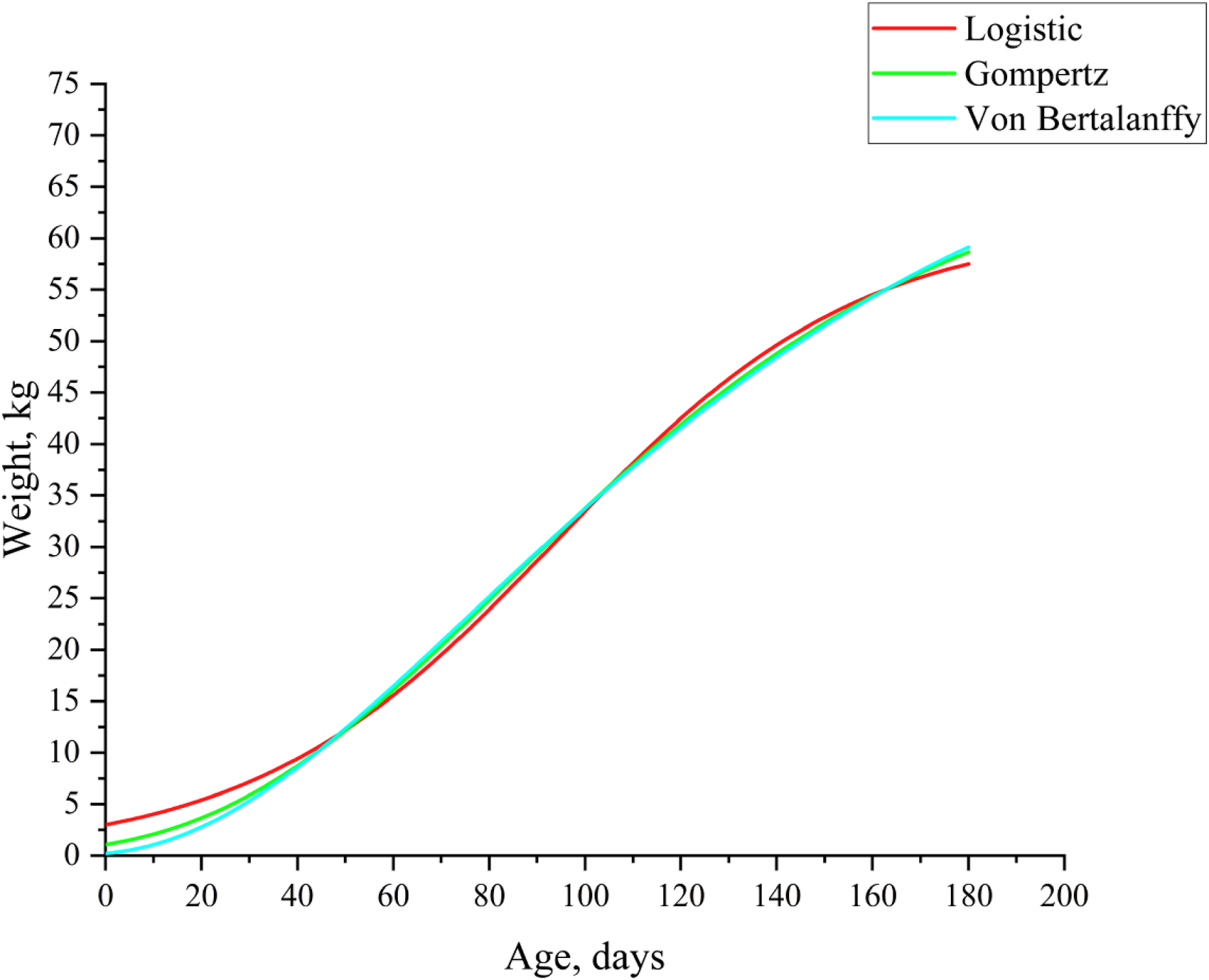
Fitting the growth curve of Neijiang pigs. Lines of different colors represent different growth curve models.

To compare the growth differences of Neijiang pigs, we used three nonlinear models to fit the growth curves of Neijiang pigs from the nursery stage to the finishing stage (0–180 days of age) and compared the predictive accuracy of the models (Table 1). Table 2 lists the parameter estimates associated with the three growth curve models. All three models fitted typical S-shaped curves, and all fitted curves were close to the observed values (Figure 1). Based on Pearson correlation coefficients and on-site measurement data, the Gompertz model was identified as the most suitable growth curve model for Neijiang pigs (*R*^2^ = 0.9961). In the optimal growth model, the growth rate of Neijiang pigs reached its peak at 84.20 days, with a body weight of 25.93 kg (Table 2).

**Table 2:** Estimates of growth curve fitting parameters.

Model	*A*	*B*	*K*	Wi , kg	Day at inflection	Maximum daily gain, g
Logistic	61.382	19.435	0.031	30.691	95.712	475.711
Gompertz	70.498	4.184	0.017	25.935	84.192	440.891
Von Bertalanffy	78.333	0.863	0.013	23.210	73.175	452.591

A,B,K , specific parameters in the function; Wi , Weight of inflection.

### 3.2 Correlation analysis of body weight and body mass index

**Figure 2:**
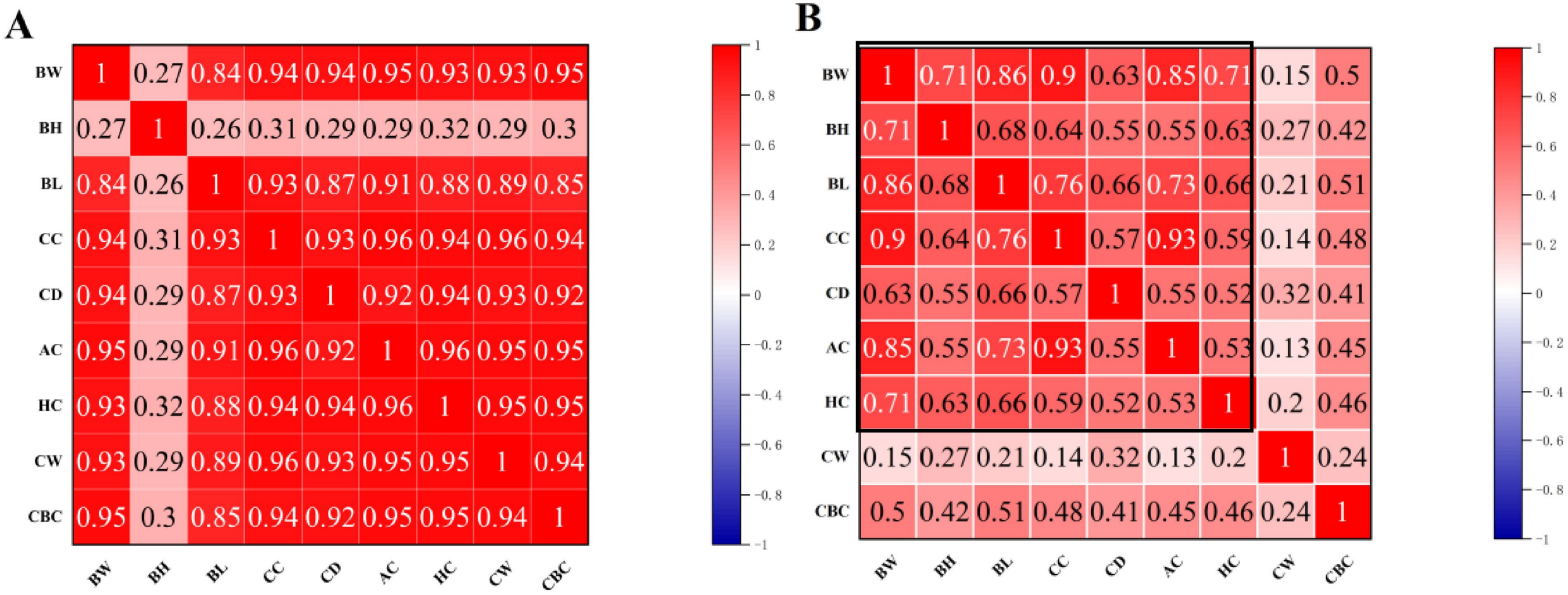
Heat map of Pearson correlation coefficient matrix. **(A)** Early growth stage (before 84 days of age); **(B)** Finishing growth stage (after 84 days of age). BW, body weight; BL, body length; BH, body height; CC, chest circumference; AC, abdominal circumference; HC, hip circumference; CW, chest width; CD, chest depth; CBC, cannon bone circumference.

Based on the optimal growth model, we divided the growth stages of Neijiang pigs into two distinct patterns according to the inflection point age: early growth stage (before 84 days of age) and finishing growth stage (after 84 days of age). Pearson correlation analysis was conducted on body conformation indicators and body weight between the early and finishing growth stages. Figure 2 shows the Pearson correlation coefficients between the indicators. In the early growth stage, the coefficient with BH is low, while the coefficients with BW, BL, BH, CC, AC, HC, and CD are all highly correlated. In the finishing growth stage, the correlation coefficients with BW, BL, BH, CC, AC, HC, and CD are all high, while the coefficients with CW and CBC are low compared to other indicators.

### 3.3 Taxonomy and diversity of gut microbiota of Neijiang pigs

**Figure 3:**
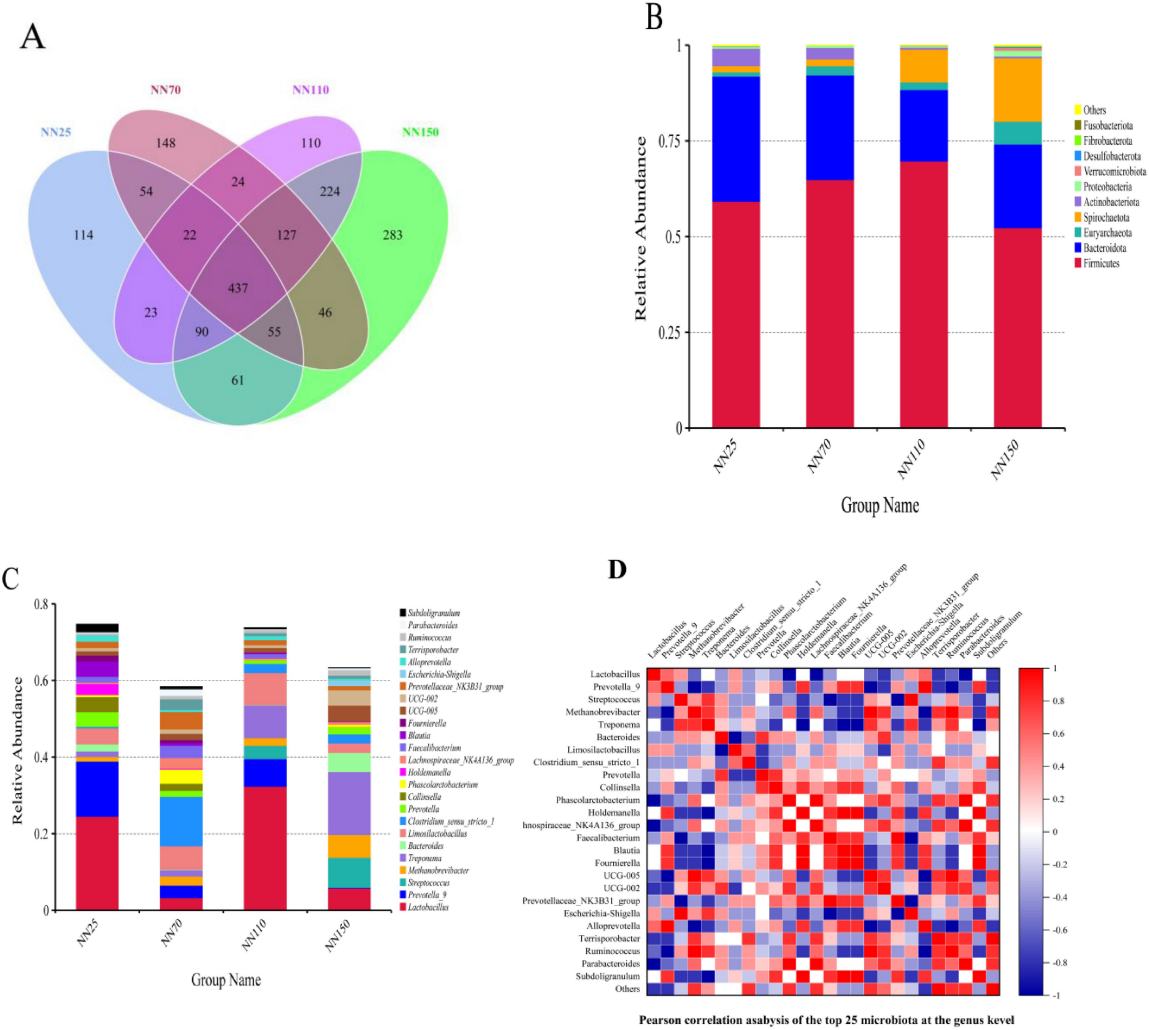
Analysis of the number of operational taxonomic units (OTUs) and the abundance of annotated species in the intestinal microbiota of Neijiang pigs at different growth stages. **(A)** Venn diagram showing the number of OTUs at different growth stages. NN25, 25-day-old Neijiang pigs; NN70, 70-day-old Neijiang pigs; NN110, 110-day-old Neijiang pigs; NN150, 150-day-old Neijiang pigs. **(B)** Histogram of relative abundance at the phylum level across different groups. **(C)** Histogram of relative abundance at the genus level across different groups. **(D)** Heatmap of Pearson correlation coefficients at the genus level.

16S rRNA sequencing was performed on Neijiang pigs at different growth stages (25, 70, 110, and 150 days old) to analyze their gut microbiota and compare differences in gut microbial composition and function. After quality control, each sample generated 2,833 valid OTUs for subsequent analysis. The α diversity analysis is shown in Table 3. Good’s coverage index was greater than 0.99 in all groups. Based on the dilution curve and abundance curve (Supplementary Figure S1), the sequencing depth represented the gut microbiome of Neijiang pigs and could therefore be used for further analysis. Figure 3A shows that the species richness and structure of the intestinal microbiota of Neijiang pigs underwent significant changes with increasing age. A total of 437 OTUs were shared among the four growth age groups, indicating the presence of a core microbiota that is widely distributed across different growth stages of Neijiang pigs. Additionally, we found that groups with similar ages shared a higher number of OTUs, while Neijiang pigs at different growth stages had a certain number of unique OTUs. For example, the 25-day-old group (NN25) had 114 unique OTUs, while the 150-day-old group (NN150) had a significantly increased number of unique OTUs, reaching 283. In all groups, Firmicutes, Bacteroidota, Euryarchaeota, Spirochaetota, Actinobacteriota, and Proteobacteria were the main six phyla (over 98% of relative abundance) (Figure 3B). The Firmicutes and Bacteroidota phyla are the two most abundant phyla across all growth stages. However, the abundance of the Spirochaetes phylum increases with age, while the abundance of the Actinobacteria phylum decreases with age (Figure 3B). At the genus level, the abundance of *Lactobacillus* was generally high, but there were differences in the abundance of *Lactobacillus* in Neijiang pigs at different growth stages. The NN25 and NN110 groups had the highest abundance, while the abundance of Lactobacillus in the other two groups decreased significantly (Figure 3C). Furthermore, the abundance of *Prevotella_9* in Neijiang pigs was higher in the early growth stage than in the later stages, which may be related to the pigs’ increased demand for rapid nutrient absorption and energy conversion during the early growth stage. In contrast, the relative abundance of *Methanobrevibacter* and *Treponema* showed an opposite trend, increasing with age, which may be associated with the pigs’ increased demand for specific metabolic products during the later growth stages (Figure 3C). *Bacteroides* abundance was significantly higher at 25 and 150 days of age compared to other stages, while *Streptococcus* abundance significantly increased in later growth stages (110 and 150 days of age), potentially reflecting changes in nutritional requirements and digestive capacity across different growth stages in Neijiang pigs. Figure 3D shows the correlations among the top 25 most abundant genera in the intestinal microbiota of Neijiang pigs based on Pearson correlation analysis.

**Table 3:** Alpha diversity of the intestinal microbiota of Neijiang pigs at different stages of growth.

Age, days	Observed species	Shannon	Simpson	Chao1	Ace	Good’s coverage	PD whole tree
NN25	295.33 ± 140.37	4.55 ± 1.05	0.8697 ± 0.0842	316.47 ± 140.26	320.97 ± 141.43	0.9994 ± 0.0002	33.46 ± 15.75
NN70	500.17 ± 38.79	6.01 ± 0.41	0.9572 ± 0.0148	525.58 ± 31.37	529.54 ± 31.80	0.9991 ± 0.0001	40.60 ± 4.44
NN110	472.83 ± 125.19	4.80 ± 0.63	0.8880 ± 0.0469	510.27 ± 134.91	510.76 ± 138.47	0.9990 ± 0.0004	39.69 ± 8.35
NN150	712.67 ± 36.18	5.82 ± 0.53	0.9277 ± 0.0340	767.79 ± 42.09	773.36 ± 41.54	0.9984 ± 0.0004	60.76 ± 6.45

The observed species index shows the number of OTUs actually observed; Shannon and Simpson indices measure biodiversity; Chao1 and Ace indices reflect the microbial species richness; Good’s coverage index shows coverage of sequencing data; PD whole tree index reflects the diversity based on the phylogenetic tree. All values are reported as means ± standard deviation (SD).

### 3.4 Differences in gut microbiota composition between different growth stages

**Figure 4:**
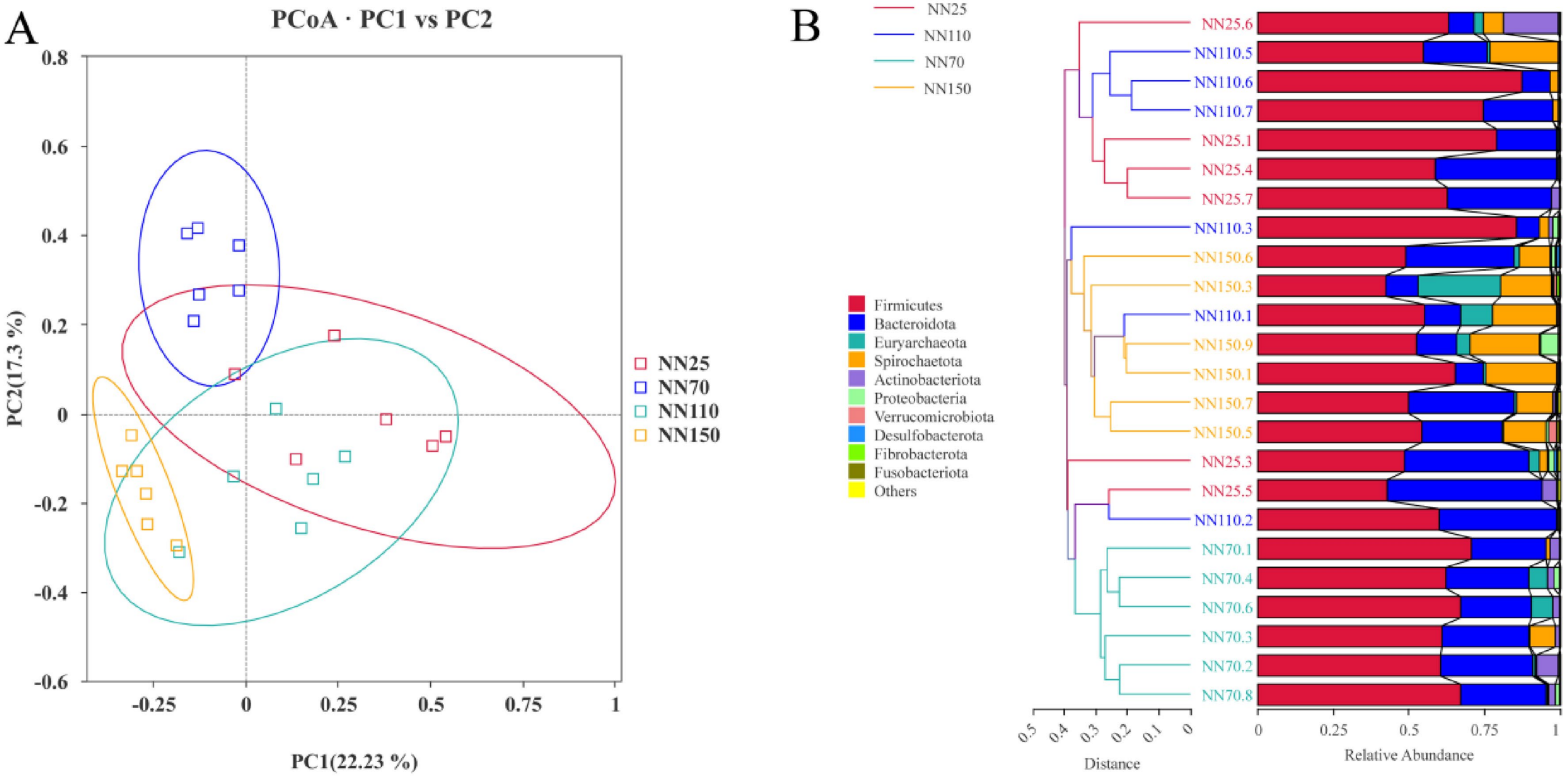
**(A)** Principal Co-ordinates Analysis (PCoA) and **(B)** unweighted pair group method with arithmetic means analysis (UPGMA) based on Bray-Curtis distances of the phylum-level relative abundance in the gut microbiota of Neijiang pigs between different growth stages.

This study further investigated the composition of the gut microbiota of Neijiang pigs at different growth stages (20 days old, 70 days old, 110 days old, and 150 days old). PCoA analysis based on Bray-Curtis distance showed that the composition of the intestinal microbiota of Neijiang pigs differed at each growth stage (Figure 4A). Clustering trees were built using UPGMA clustering based on Bray-Curtis distances matrices (Figure 4B), and the results showed that the gut microbiota composition at different growth stages formed independent clusters. Notably, in both PCoA and UPGMA analyses, the gut microbiota composition at the terminal growth stages (110 days and 150 days) was more similar than that at the early growth stages (20 days and 70 days).

**Figure 5:**
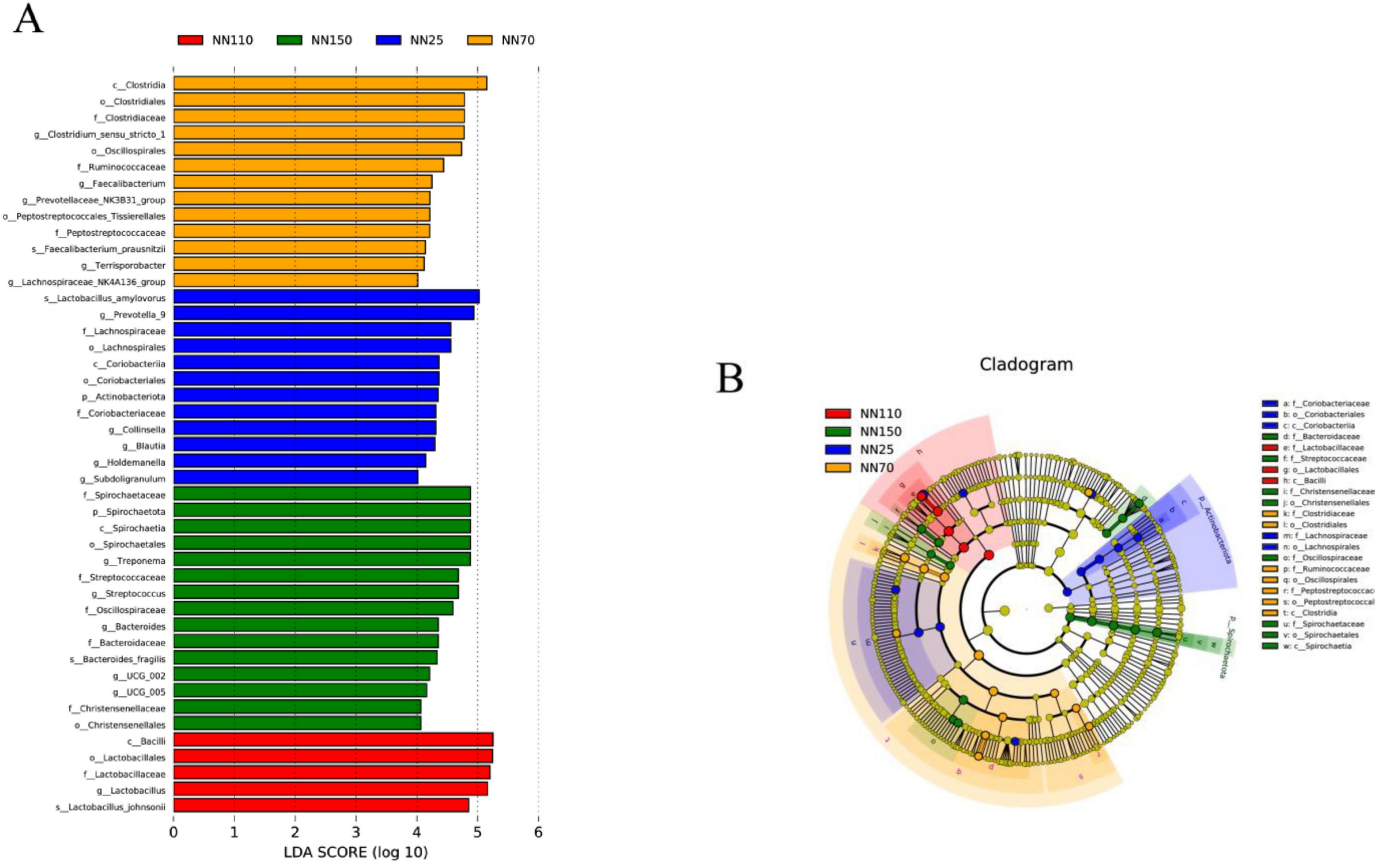
**(A,B)** Differences in the gut microbiota composition of different growth Stages based on Linear discriminant analysis effect size (LEfSe, LDA > 4).

In addition, LEfSe analysis was performed to determine the abundance of specific microbial taxa at different growth stages. A LDA score (log_10_) greater than four was considered the threshold. A total of 45 potential biomarkers were identified across all growth stages of the Neijiang pigs (12 in the NN25 group; 13 in the NN70 group; 5 in the NN110 group; and 15 in the NN150 group) (Figure 5). At 25 days of age, *Prevotella_9*, *Collinsella*, and *Blautia* are the most abundant genera in the intestinal microbiota. Among these, *Prevotella_9* serves as a potential marker for the intestinal microbiota at this stage and may play a crucial role in helping piglets adapt to solid feed after weaning by breaking down complex carbohydrates to enhance nutrient absorption; *Collinsella* and *Blautia* may be associated with maintaining gut microbiota balance and host health, although their specific functions require further investigation. *Clostridium_sensu_stricto_1*, *Faecalibacterium*, and *Prevotellaceae_ NK3B31_group* are the most abundant genera at 70 days of age. Among these, *Faecalibacterium*, as a known butyrate-producing bacterium, plays an important role in maintaining intestinal barrier function and immune regulation; *Clostridium_sensu_stricto_1* and *Prevotellaceae NK3B31* group may be involved in the degradation of cellulose and polysaccharides in the gut, aiding the host in obtaining energy from plant-based feed. *Lactobacillus* is the most abundant genus at 110 days of age. This genus is typically considered a probiotic, inhibiting pathogen growth by producing lactic acid and other metabolic products, enhancing host immunity, and positively influencing intestinal health. At 150 days of age, *Treponema*, *Streptococcus*, and *Bacteroides* become the dominant genera. *Bacteroides* exerts significant effects on host intestinal health and energy balance through the production of short-chain fatty acids; *Treponema* and *Streptococcus* may be associated with host immune regulation and maintenance of intestinal health.

### 3.5 Comparison of the functions of gut microbiota at different stages of growth

**Figure 6:**
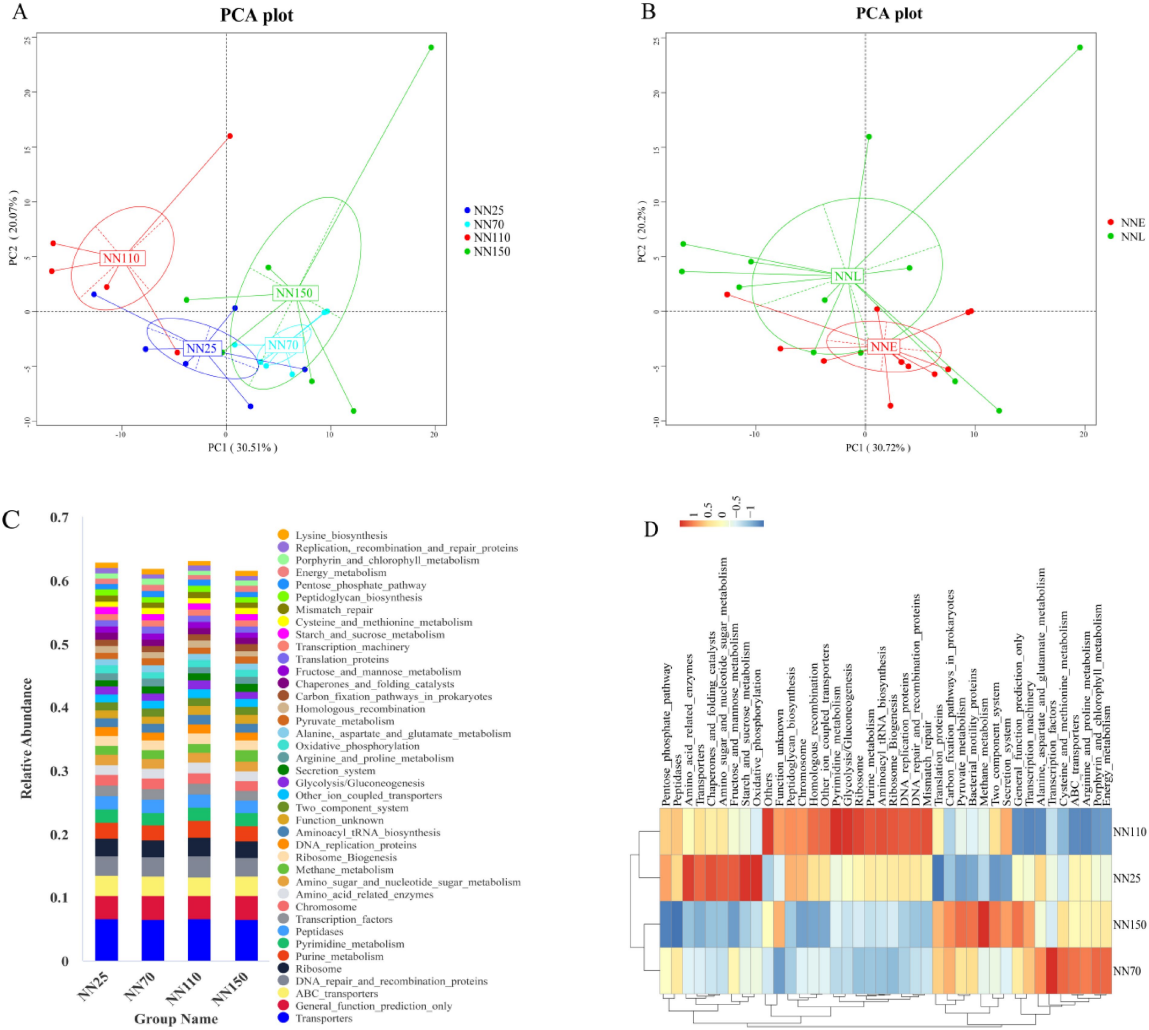
KEGG pathway analysis based on PICRUSt2 predicts the functional composition of the gut microbiota in Neijiang pigs at different growth stages. **(A,B)** Principal component analysis of KEGG pathway abundance. NN25, 25 days old; NN70, 70 days old; NN110, 110 days old; NN150, 150 days old; NNE, early growth stage (25 and 70 days old); NNL, finishing growth stage (110 and 150 days old). **(C)** Histogram of KEGG pathway abundance between different sample groups. **(D)** Clustering heatmap of KEGG pathway abundance across different sample groups.

It was found that the gut microbiota composition of Neijiang pigs varied significantly across different growth stages. To further explore the functional implications of these compositional changes, metagenomic functions were predicted using PICRUSt2 based on the KEGG pathway database. Predicted functional richness was used to generate a Principal Component Analysis (PCA) plot, which revealed distinct clustering patterns among samples (Figure 6A). Notably, samples from stages NN25 and NN70 clustered closely along PC1, indicating similar microbial functional profiles during early growth. In contrast, NN110 and NN150 were clearly separated, particularly along PC1, suggesting a significant functional divergence in finishing growth stages. To better illustrate these differences, NN25 and NN70 were combined into the early growth group (NNE), and NN110 and NN150 into the finishing growth group (NNL). PCA based on these groupings further highlighted the functional distinction between early and finishing growth stages, with clear separation along PC1 (Figure 6B).

To identify the specific functional pathways driving these differences, KEGG pathway abundance was analyzed (Figure 6C). Core functions such as “Transporters,” “General function prediction only,” “ABC transporters,” “DNA repair and recombination proteins,” and “Ribosome” were consistently abundant across all stages. Interestingly, “Methane metabolism” showed a slight increase in NN150, suggesting elevated methanogenic activity during the late finishing stage.

To further investigate the relationships among functional profiles across stages, hierarchical clustering of KEGG pathway abundances was performed (Figure 6D). The results showed that NN25 and NN110 clustered together, while NN70 and NN150 formed a separate cluster. NN25 and NN110 were enriched in pathways related to DNA repair, ribosome biogenesis, and purine metabolism, whereas NN70 and NN150 exhibited higher abundances of ABC transporters, Energy metabolism, and transcription factors. These patterns suggest dynamic and stage-specific functional adaptations. From NN25 to NN70, microbial functions shifted from basal metabolism toward nutrient transport and protein metabolism. From NN70 to NN110, there was a partial reversion to basal metabolic functions. Finally, from NN110 to NN150, functions again transitioned toward nutrient transport and protein metabolism. These transitions may reflect microbial community adaptation to changing host physiological conditions and dietary substrates during growth.

**Figure 7:**
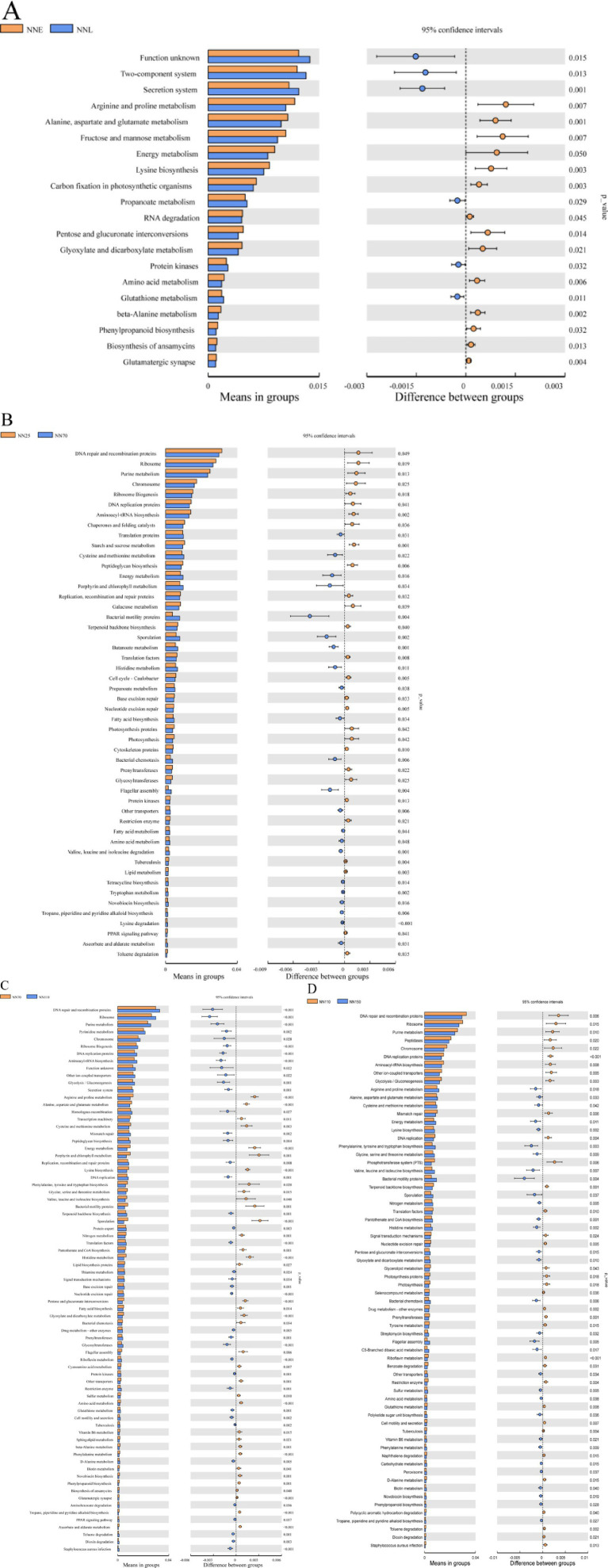
Differences in the abundance of annotated KEGG levels (level 3) in the gut microbiota composition of Neijiang pigs at different growth stages based on *T*-test analysis. **(A)** NNE vs. NNL; **(B)** NN25 vs. NN70; **(C)** NN70 vs. NN110; **(D)** NN110 vs. NN150. NNE, early growth stage (25 and 70 days old); NNL, finishing growth stage (110 and 150 days old); NN25, 25 days old; NN70, 70 days old; NN110, 110 days old; NN150, 150 days old. Extended error line plots of the significant differences between the different stages, with corrected *p*-values shown on the right.

Then, a *t*-test analysis was performed on the abundance of gut microbiota composition annotations at the KEGG level (level 3) between the early growth stage and the finishing growth stage, as well as between adjacent growth stages (Figure 7). The results showed that there were significant differences in multiple KEGG pathway functions between the early stage (NNE) and the finishing growth stage (NNL) (*p* < 0.05). Among these, the significantly enriched functional pathways in the finishing growth stage included methane metabolism, bacterial motility proteins, secretion system, two-component system, and oxidative phosphorylation. Upon further comparison of functional differences among the four stages, the differences between NN25 and NN70 were relatively minor, primarily concentrated in basic functions such as DNA repair, ribosomal function, and amino acid metabolism; the differences between NN70 and NN110 were significant, involving DNA repair, secretion system, and bacterial motility proteins; and the differences between NN110 and NN150 further expanded, with significant enhancements in functions such as methane metabolism, bacterial motility proteins, and secretion system.

## 4 Discussion

This study used the Logistic, Gompertz, and Von Bertalanffy growth curves to fit the body weight of Neijiang pigs from birth to 180 days of age. The analysis was conducted using Pearson correlation coefficients and actual body weight data measured on-site. The results indicated that the Gompertz model is the most suitable growth curve model for Neijiang pigs. In the optimal feeding model, the growth rate of Neijiang pigs reaches its peak at 84.2 days, with a body weight of 25.93 kg. Comparable outcomes (Chen et al., 2023) indicate that the Gompertz model likewise offers the optimal description of Neijiang-pig growth; nevertheless, the estimated age and weight at the inflection point differ slightly. Besides genetic background, differences in diet, external environment, feeding management, and model accuracy may lead to variations in the estimated growth inflection points related to weight.

Additionally, based on the inflection point age (approximately 84 days), the growth process of Neijiang pigs was divided into early growth and finishing stages, and the relationship between body conformation indices and body weight was further explored at different stages. During the early growth stage, the chest width (CW) and cannon bone circumference (CBC) of Neijiang pigs showed a high positive correlation with body weight, indicating that pigs in this stage are in a period of rapid skeletal and muscular development, and body conformation indicators can effectively reflect their growth status; however, body height (BH) contributed less, possibly due to incomplete skeletal maturation at this stage. Upon entering the finishing stage, the correlation between CW and body weight significantly decreased, while the correlation between BH and body weight increased, and the correlation between CBC and body weight also decreased. This may be related to pigs gradually entering the fat deposition stage, skeletal development tending toward maturity, and slowed muscle growth rates. It is worth noting that this pattern of changes in the correlation between body conformation indices and body weight is closely related to the genetic background, growth and development characteristics, and body conformation features of the Neijiang pig, a specific breed. As a local pig breed, the Neijiang pig has characteristics such as early fat deposition and a longer duration of skeletal development (Tecku et al., 2025).

The interaction between the host and the gut microbiota spans the entire lifespan of an animal. The composition of the gut microbiota varies among individuals and influences health (Kundu et al., 2017). During the growth and development of pigs, the maximum growth rate is estimated at the growth inflection point, indicating that metabolic and digestive functions are active. This study determined the gut microbiota composition and its predictive functions in Neijiang pigs at different growth stages (25 days, 70 days, 110 days, and 150 days) to describe age-based longitudinal variation. First, we explored gut microbiota diversity at different growth stages. Overall, the number of OTUs identified in Neijiang pigs at different growth stages generally increased with age (NN25 = 295.33 ± 140.37, NN70 = 500.17 ± 38.79, NN110 = 472.83 ± 125.19, and NN150 = 712.67 ± 36.18), and gut microbiota diversity increased with age, as confirmed by the observed increases in species richness, Shannon index, Chao1 index, and Ace index. As previously reported, gut microbiota diversity in pigs increases with age (Shao et al., 2021). In fact, the gut bacterial diversity of 70-day-old pigs from Neijiang was slightly higher than that of 110-day-old pigs, possibly because the 70-day-old pigs were closer to the growth inflection point stage, with more active metabolic and digestive functions. Further in-depth research is needed to confirm this. Studies have shown that there are differences in the increase and decrease of gut microbial diversity across different growth stages. The Firmicutes and Bacteroidetes phyla are the two dominant phyla in pig gut microbiota, accounting for 74–92% of the relative abundance, consistent with the results of multiple other studies (Shao et al., 2021; Kim et al., 2015; Kumar et al., 2019), but the proportions fluctuate with age. At the genus level, genera such as *Lactobacillus*, *Treponema*, and *Methanobrevibacter* exhibit significant changes across different stages, which may be closely related to factors such as diet, intestinal environment, and immune status. As Neijiang pigs age, their gut microbiota undergoes clear phasic shifts. In the early stage (NN25), Firmicutes and Bacteroidota dominate, with *Lactobacillus* and *Prevotella_9* as the main genera, likely due to colostrum intake and a stable intestinal environment. In the middle stage (NN70), weaning and dietary changes lead to increased microbial diversity, a rise in Firmicutes, and a decline in Bacteroidota, reflecting adaptation to plant-based feed. By the late middle stage (NN110), Firmicutes peaks and *Lactobacillus* re-emerges as dominant, indicating improved dietary adaptation and microbiota stability. In the late stage (NN150), Spirochaetota and Euryarchaeota significantly increase, with *Treponema* and *Methanobrevibacter* becoming dominant—possibly linked to higher fiber intake and enhanced gut anaerobiosis. Actinobacteriota, abundant early on, declines sharply with age, suggesting a role in early gut colonization. Overall, Firmicutes initially increases and then decreases, Bacteroidota shows a declining trend, while Spirochaetota and Euryarchaeota increase with age, consistent with findings from studies on humans and pigs (Kim et al., 2015). These patterns reflect the dynamic adaptation of the gut microbiome in response to weaning, diet, environmental changes, and immune development.

To explore stage-specific differences in gut microbiota, PCoA and UPGMA clustering were performed based on Bray-Curtis distance. Results showed clear separation among microbial communities across growth stages, particularly between NN25 and NN150, indicating that host age is a major driver of microbial dynamics. Partial overlap between NN70 and NN110 suggested a transitional profile, likely due to dietary shifts or individual variation. UPGMA clustering largely supported this pattern, though some cross-stage clustering indicated environmental or host-specific influences. LEfSe analysis identified stage-specific biomarkers. At NN25, *Prevotella_9*, *Collinsella*, and *Blautia* were enriched, potentially aiding early adaptation to solid feed and maintaining gut homeostasis, so it is recommended to increase the amount of easily digestible carbohydrates and protein sources in the diet during the early growth stage, while adding prebiotics such as fructooligosaccharides (FOS) to promote the growth of beneficial bacteria, enhance intestinal health, and strengthen the immune function of piglets. At NN70, *Faecalibacterium*, *Clostridium_sensu_stricto _1*, and *Prevotellaceae_NK3B31_group* were dominant, reflecting metabolic adaptation to plant-based diets. It is recommended to appropriately increase the cellulose content in the daily diet during this stage, while adding an appropriate amount of oil to increase energy density and meet the energy requirements for rapid growth in pigs. At NN110, *Lactobacillus*, *Streptococcus*, and *Treponema* were abundant, indicating enhanced fermentation capacity and immune modulation. At NN150, *Prevotella_9*, *UCG_002*, and *Treponema* were enriched, suggesting adaptation to high-fiber diets. Therefore, it is recommended to further increase the cellulose content in the diet during the finishing growth stages while reducing protein levels to reduce nitrogen emissions and improve feed conversion efficiency. In addition, methane inhibitors such as nitrate or 3-NOP can be considered to reduce methane production and further improve feed energy utilization. It is worth noting that the LEfSe analysis results largely validated our previous abundance trends at the door and genus levels, which remained consistent in the NN110 and NN150 stages. This also reflects that the intestinal microbiota of Neijiang pigs is more stable in the middle and late stages of growth. However, the inconsistency of some specific microbiota may be attributed to differences in analysis methods, statistical thresholds, or sample individual differences. These findings align with recent studies on age-related microbial succession in pigs and humans (Ma et al., 2022; Zhou et al., 2025; Chen et al., 2021; Johnson et al., 2019). Future metagenomic studies are warranted to elucidate the functional roles of these taxa and inform targeted nutritional strategies for improving gut health in local pig breeds.

Finally, KEGG pathway analysis was performed based on PICRUSt2 to predict the functions of Neijiang pig intestinal microbiota at different growth stages. PCA and clustering analyses revealed stage-specific microbial functions, with distinct transitions between early and finishing growth. The close clustering of NN25 and NN70 in PCA suggests functional stability during early development. In contrast, the divergence of NN110 and NN150 indicates significant functional remodeling in later stages, likely reflecting shifts in host nutrient requirements and intestinal physiology.

The relative abundance of core KEGG pathways was consistent across stages, highlighting the importance of basic metabolic and transport functions. The slight increase in ‘Methane metabolism’ in NN150 may reflect changes in the gut environment during late finishing, possibly due to diet composition or microbial community shifts. Interestingly, hierarchical clustering revealed a different pattern: NN25 grouped with NN110, and NN70 with NN150. This discrepancy suggests that functional similarity depends on the analytical perspective. At the NN25 stage, intestinal microbial function is primarily characterized by high expression of basal metabolic pathways, which aligns with the physiological requirements of early rapid growth and vigorous cell proliferation in piglets. Upon entering the NN70 stage, intestinal microbial function gradually shifts toward pathways related to nutrient transport and protein metabolism, which may be associated with accelerated growth rates and increased nutrient requirements during the mid-growth phase of piglets. Notably, the NN110 stage again exhibits high expression of basal metabolic pathways, suggesting a potential functional reversion or transition during the mid-growth phase. This reversion may be associated with adjustments in the host’s physiological state or changes in the intestinal environment. Upon entering the NN150 stage, intestinal microbial function again shifts toward pathways related to nutrient transport and protein metabolism, aligning with the increased demand for energy storage and protein deposition in the late finishing phase. These functional pattern changes between adjacent stages suggest that the composition of gut microbial functions may exhibit a certain degree of transitional characteristics across different growth stages. This transitional nature may be closely related to changes in the host’s physiological requirements across different growth stages, as well as gradual changes in the gut environment (e.g., pH, oxygen concentration, nutrient composition). Aligning predicted pathway abundances with growth curve inflection points reveals that even sub-percent Level-3 module changes carry physiological significance. The continuous gradient from “ABC transporters → fructose and mannose metabolism → pyruvate metabolism” during the NN25 phase provides precursors for microbial butyrate synthesis, coinciding with the window when the colonic barrier matures and “energy metabolism” accounts for the lowest proportion. At the mathematically defined inflection point (NN70), the peak “lysine biosynthesis” pathway (0.87%) diverts reducing equivalents from hydrogen-dependent methane production by consuming NADPH, delaying the rise of “Methane metabolism” until the finishing phase. Subsequently, a 0.45%-unit increase in methane pathways represents additional digestible energy loss, whose temporal trajectory perfectly aligns with the post-inflection point deceleration in daily weight gain. Thus, PICRUSt2-predicted functional shifts centered on the gut microbiome quantitatively explain the physiological switch from accelerated to decelerated growth in Neijiang pigs. T-test analysis further clarified the dynamic process of microbial community functional changes.

Although this study used age as the primary variable under standardized Neijiang pig rearing conditions, it did not experimentally manipulate management factors such as manure removal frequency, fermented feed supply, or intermittent flushing—all variables capable of rapidly altering microbial metabolism. Although these factors were standardized in this trial (daily manure removal, non-fermented dry feed, high-pressure flushing between batches), future studies should employ factorial designs to systematically compare the effects of alternate-day vs. daily manure removal or fermented feed vs. dry feed within the same age cohort. This approach would distinguish management effects from inherent developmental trajectories and refine the microecological intervention window specific to this local breed.

## 5 Conclusion

This study analyzed growth dynamics and gut microbiota development in Neijiang pigs from birth to 180 days. The Gompertz model best described the growth curve, with an inflection point at 84.2 days (25.93 kg). Early growth was characterized by rapid skeletal and muscular development, whereas the finishing stage showed increased fat deposition and skeletal maturation. Gut microbiota composition and function changed significantly with age across four stages (25, 70, 110, and 150 days). Microbial diversity increased over time, with distinct shifts in dominant taxa and stage-specific biomarkers identified. Functional profiles revealed dynamic adaptations, with core metabolic pathways consistently abundant. Notably, hierarchical clustering indicated potential functional reprogramming or transition during the intermediate stage (NN70–NN110), highlighting the microbiota’s adaptability to host physiological changes and intestinal environment fluctuations. These findings underscore the dynamic interplay between host growth and gut microbiota development, offering insights for optimizing feeding strategies and improving production efficiency in Neijiang pigs.

Within the standardized Neijiang pig system, this study proposes two low-cost, farm-verifiable application strategies: (i) moderately reducing dietary crude protein while supplementing crystalline L-lysine to exploit microbial lysine peaks; (ii) adding triglyceride butyrate or sodium butyrate during the first 2 weeks post-weaning to potentially enhance endogenous butyrate flux. Looking ahead, future research should: - Deepen exploration of the revealed host-microbiome mechanisms by concurrently validating “lysine synthesis-nitrogen conservation” through ileal transcriptome and microbial metagenome analyses; Establishing cross-stage fecal microbiota transplantation cycles to evaluate whether “early introduction of late-stage bacteria” accelerates barrier maturation or reduces maintenance energy expenditure. These mechanism-driven studies will further elucidate the coupling patterns between microbial function and growth performance in this local breed.

## Data Availability

The data presented in this study are publicly available. The data can be found here: https://www.ncbi.nlm.nih.gov/sra, accession PRJNA1354224.

## Footnotes

^1^ http://ccb.jhu.edu/software/FLASH/

^2^ https://www.arb-silva.de/

^3^ https://unite.ut.ee/

^4^ https://github.com/torognes/vsearch

^5^ http://www.drive5.com/uparse/
