# Supplementary material for: Age-related gut microbiota succession in Neijiang pigs: insights for precision feeding and productivity
Source: Front Microbiol. 2025 Nov 12;16:1698169. doi: 10.3389/fmicb.2025.1698169 (PMC12651433; doi:10.3389/fmicb.2025.1698169)
Supplement: Supplementary file 1 [file Supplementary_file_1.docx]

Supplementary Material

# Supplementary Figures


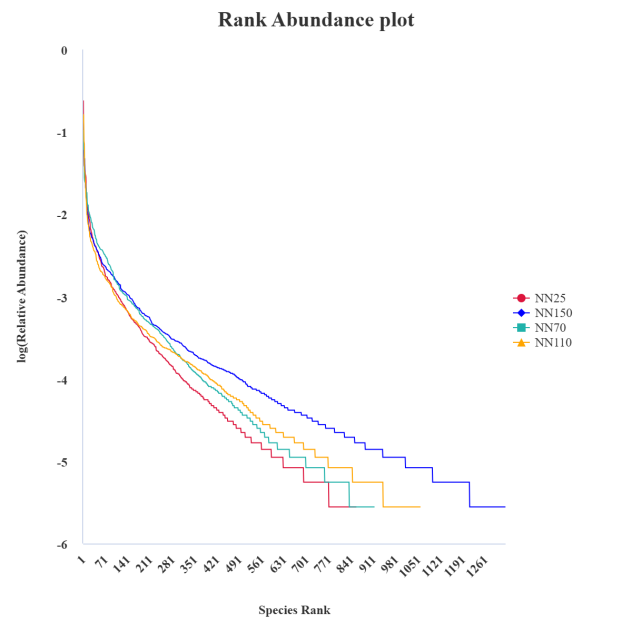

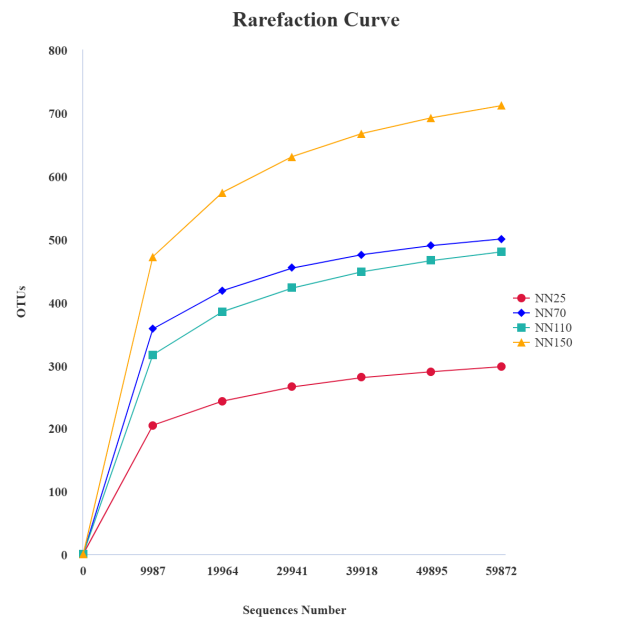


**Figure S1.** The rarefaction curve and abundance curve.


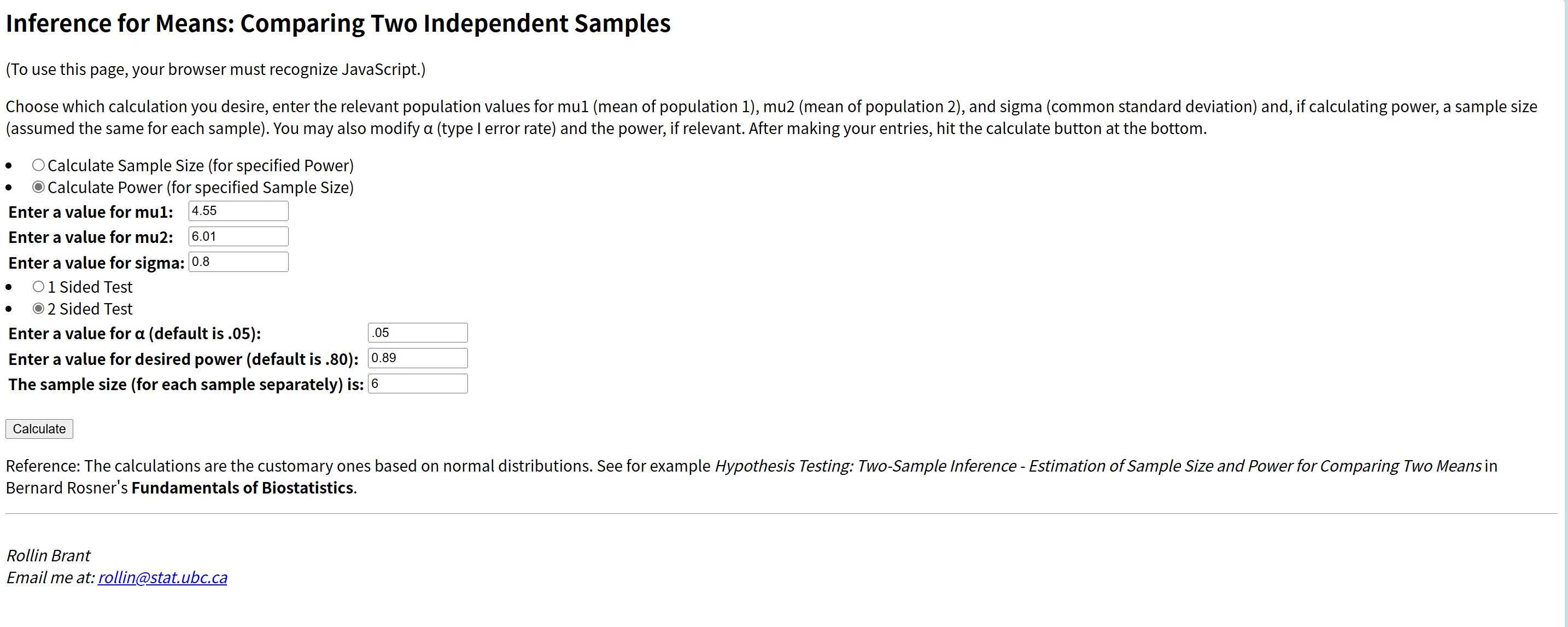


**Figure S2.** A post-hoc power analysis (Shannon index, 25 d vs. 70 d, most conservative; mean difference = 1.46, pooled SD = 0.80, α = 0.05, two-tailed)


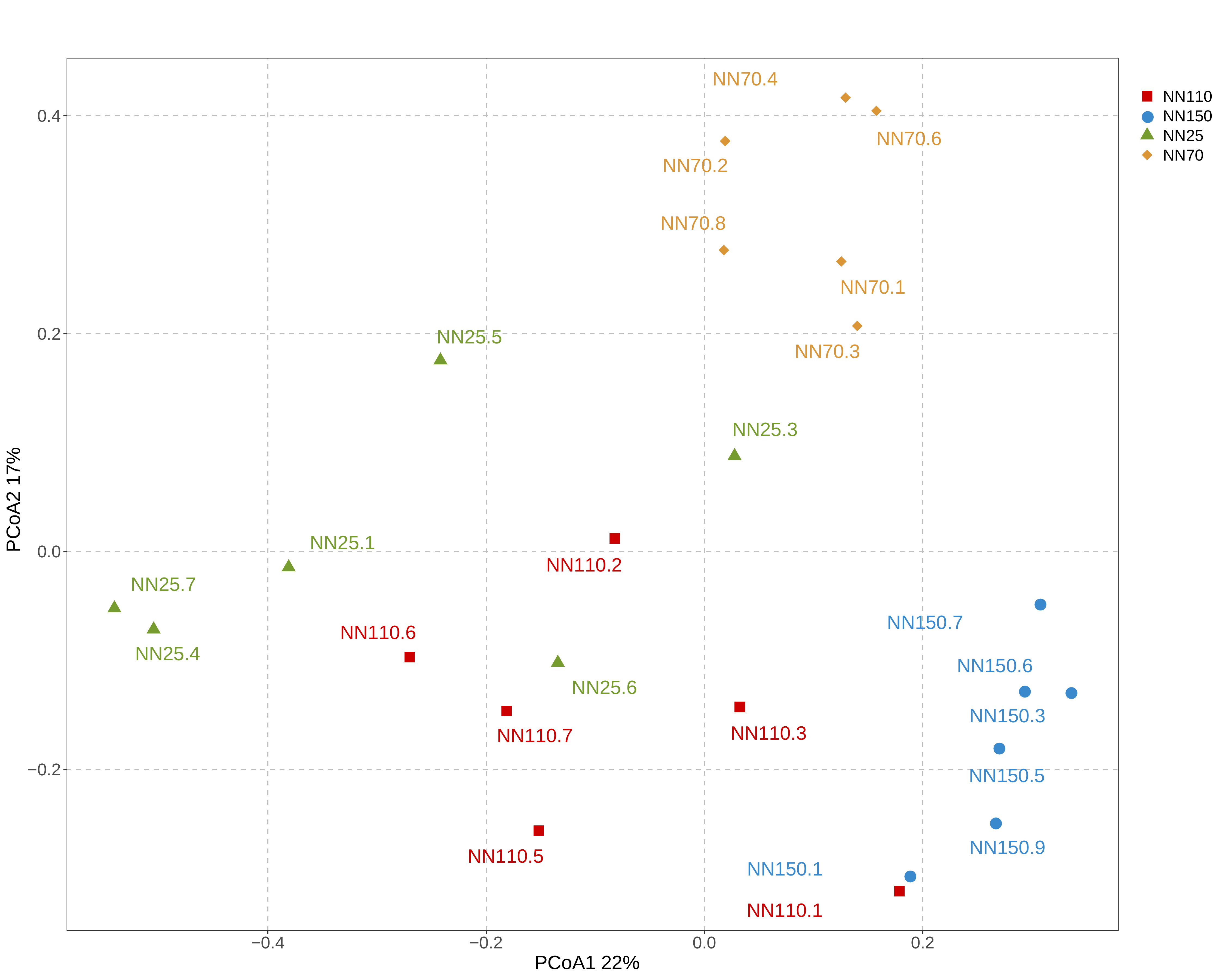

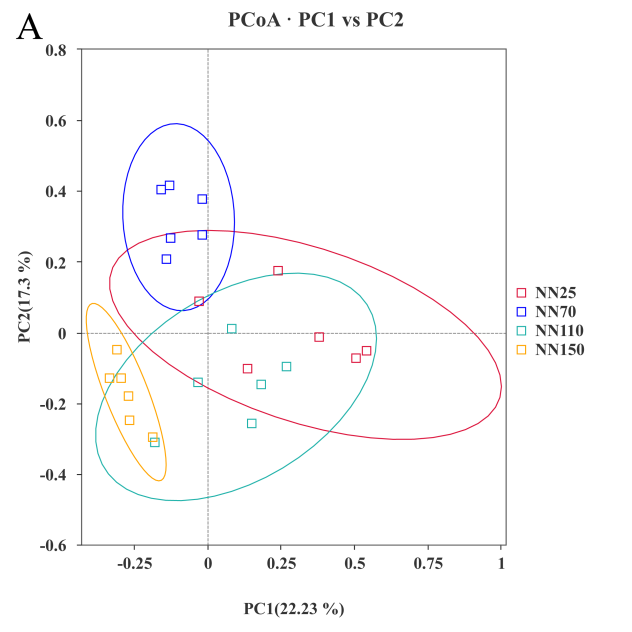


**Figure S3.** QIIME 2 vs Original Results Comparison

| Qiime2 | Age，days | Observed Species | Shannon | Simpson | Chao1 | Ace | Good’s Coverage |
| --- | --- | --- | --- | --- | --- | --- | --- |
|  | NN25 | 297±141.12 | 3.33±0.43 | 0.8697±0.0842 | 317.94±144.72 | 322.49±145.08 | 0.9994±0.0002 |
|  | NN70 | 499.67±41.73 | 4.03±0.37 | 0.9572±0.0148 | 528.41±30.66 | 531.21±34.80 | 0.9991±0.0001 |
|  | NN110 | 479.17±125.64 | 3.15±0.73 | 0.8880±0.0469 | 529.33±138.79 | 527.19±137.79 | 0.9990±0.0004 |
|  | NN150 | 711±32.25 | 4.16±0.29 | 0.9277±0.0340 | 769.37±36.27 | 771.38±30.54 | 0.9984±0.0004 |
|  |  |  |  |  |  |  |  |
| Original text | Age，days | Observed Species | Shannon | Simpson | Chao1 | Ace | Good’s Coverage |
|  | NN25 | 295.33±140.37 | 4.55±1.05 | 0.8697±0.0842 | 316.47±140.26 | 320.97±141.43 | 0.9994±0.0002 |
|  | NN70 | 500.17±38.79 | 6.01±0.41 | 0.9572±0.0148 | 525.58±31.37 | 529.54±31.80 | 0.9991±0.0001 |
|  | NN110 | 472.83±125.19 | 4.80±0.63 | 0.8880±0.0469 | 510.27±134.91 | 510.76±138.47 | 0.9990±0.0004 |
|  | NN150 | 712.67±36.18 | 5.82±0.53 | 0.9277±0.0340 | 767.79±42.09 | 773.36±41.54 | 0.9984±0.0004 |

**Table S1.** QIIME 2 vs Original Results Comparison





**Figure 1.** Fitting the growth curve of Neijiang pigs. Lines of different colors represent different growth curve models.


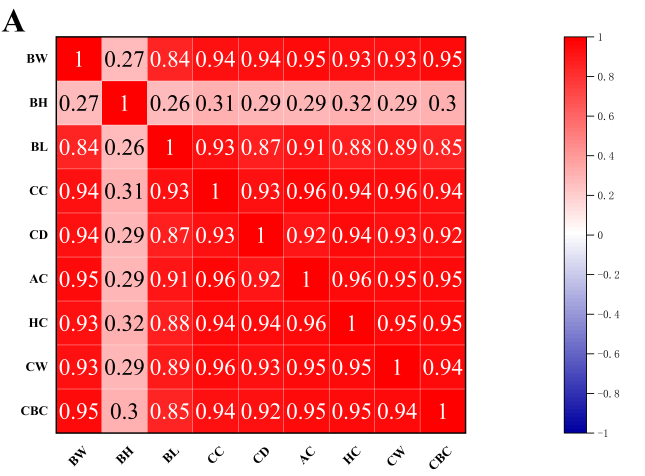

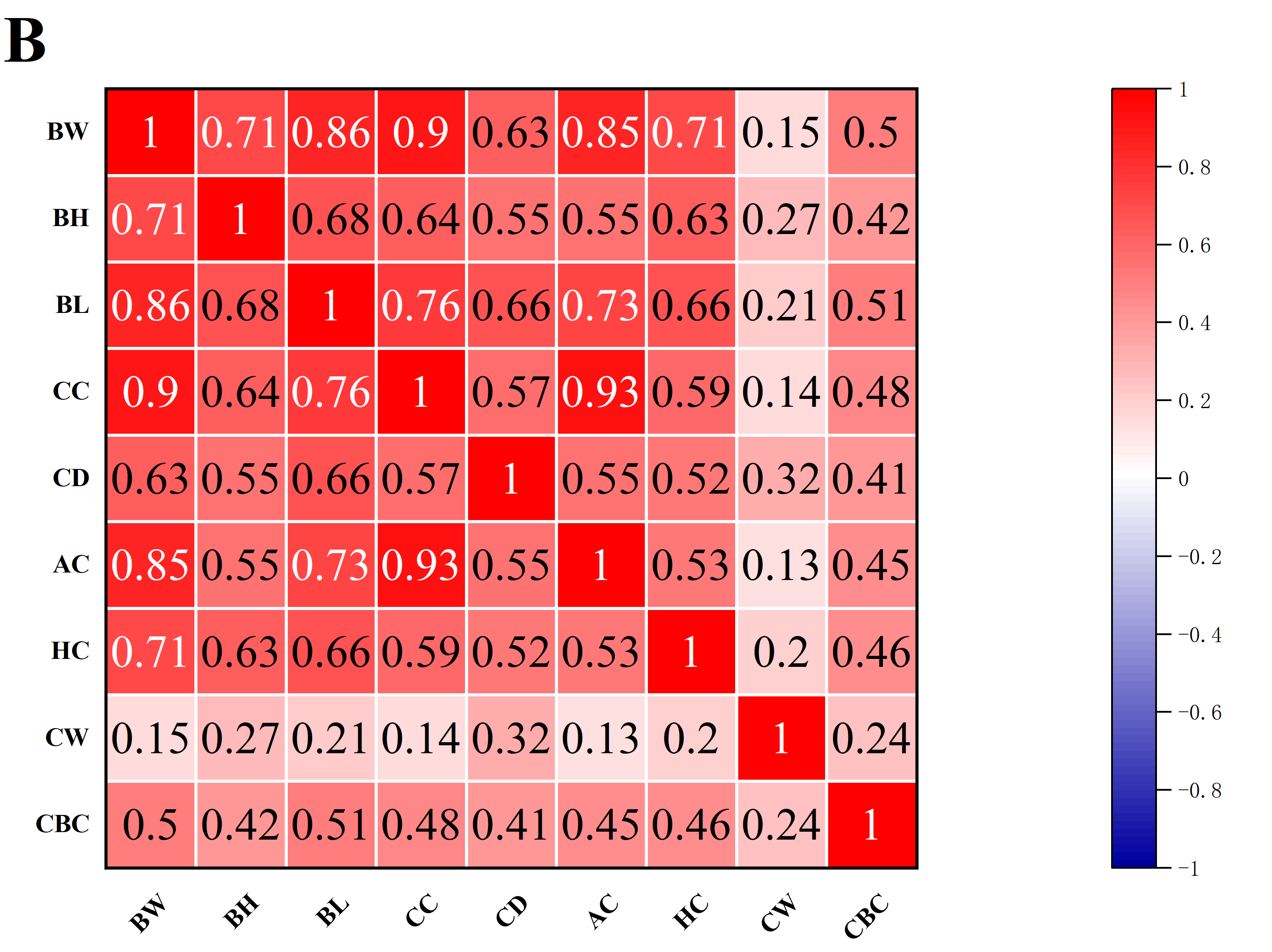


**Figure 2.** Heat map of Pearson correlation coefficient matrix. (A) Early growth stage (before 84 days of age); (B) Finishing growth stage (after 84 days of age). BW—body weight; BL—body length; BH—body height; CC—chest circumference; AC—abdominal circumference, HC—hip circumference; CW—chest width; CD—chest depth; CBC—cannon bone circumference.


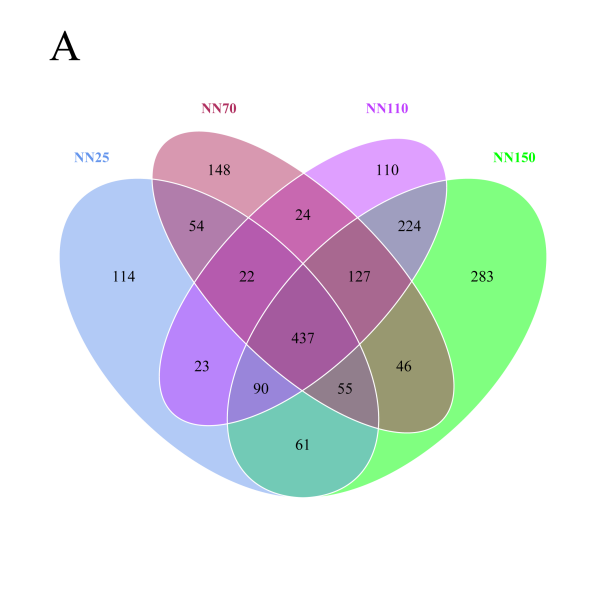

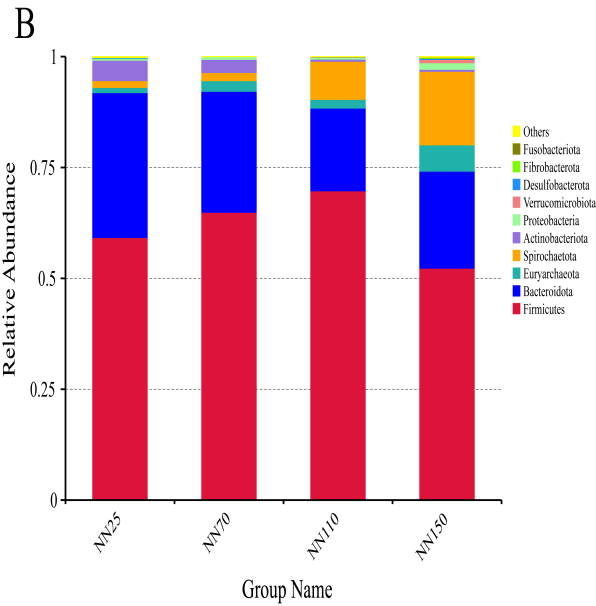


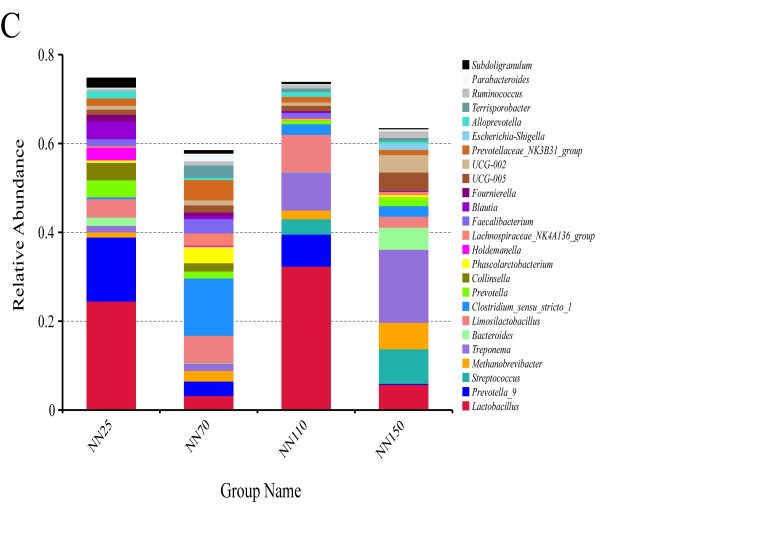

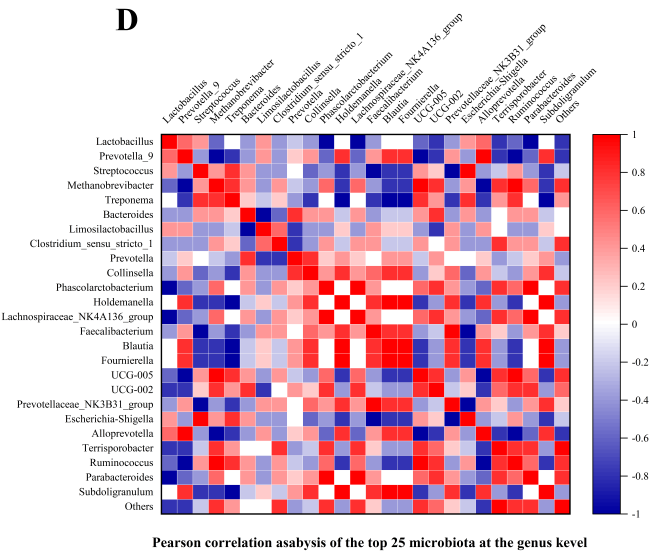


**Figure 3.** Analysis of the number of operational taxonomic units (OTUs) and the abundance of annotated species in the intestinal microbiota of Neijiang pigs at different growth stages. (A) Venn diagram showing the number of OTUs at different growth stages. NN25—25-day-old Neijiang pigs; NN70—70-day-old Neijiang pigs; NN110—110-day-old Neijiang pigs; NN150—150-day-old Neijiang pigs. (B) Histogram of relative abundance at the phylum level across different groups. (C) Histogram of relative abundance at the genus level across different groups. (D) Heatmap of Pearson correlation coefficients at the genus level.


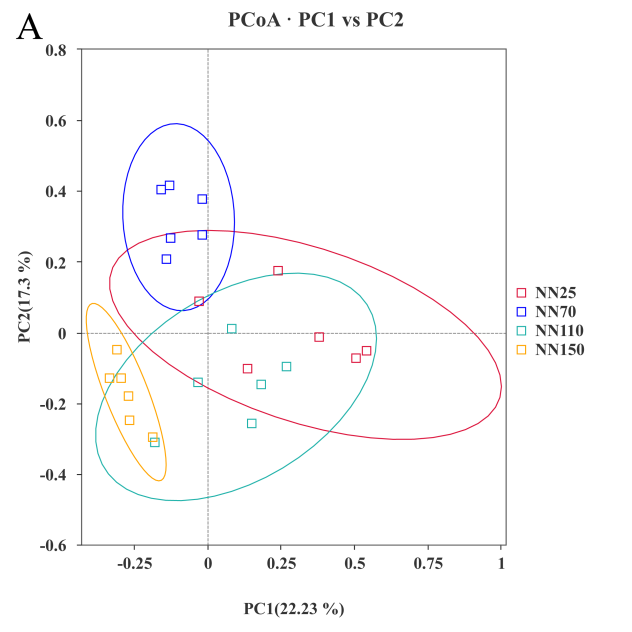

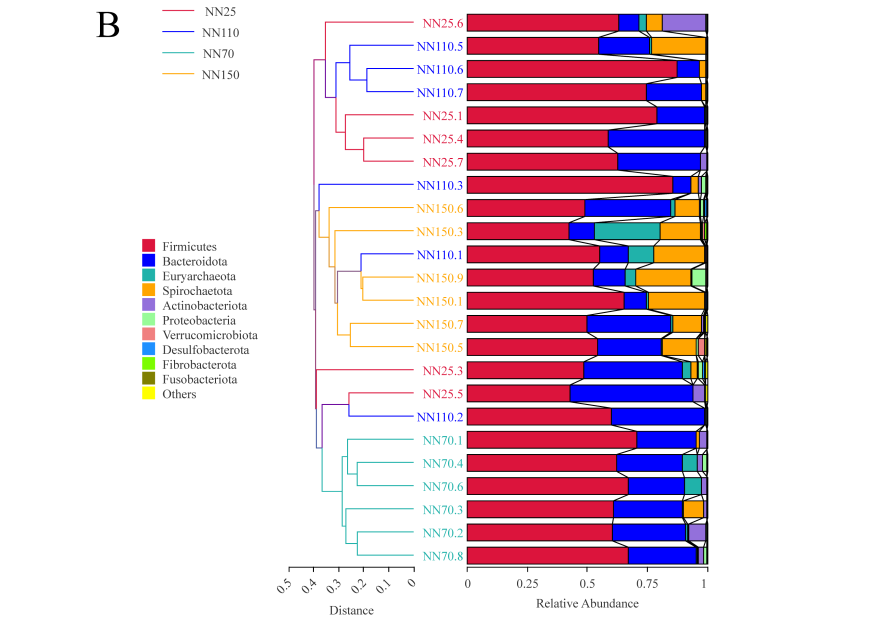


**Figure 4.** (A) Principal Co-ordinates Analysis (PCoA) and (B) Unweighted pair group method with arithmetic means analysis (UPGMA) based on Bray-Curtis distances of the phylum-level relative abundance in the gut microbiota of Neijiang pigs between different growth stages.


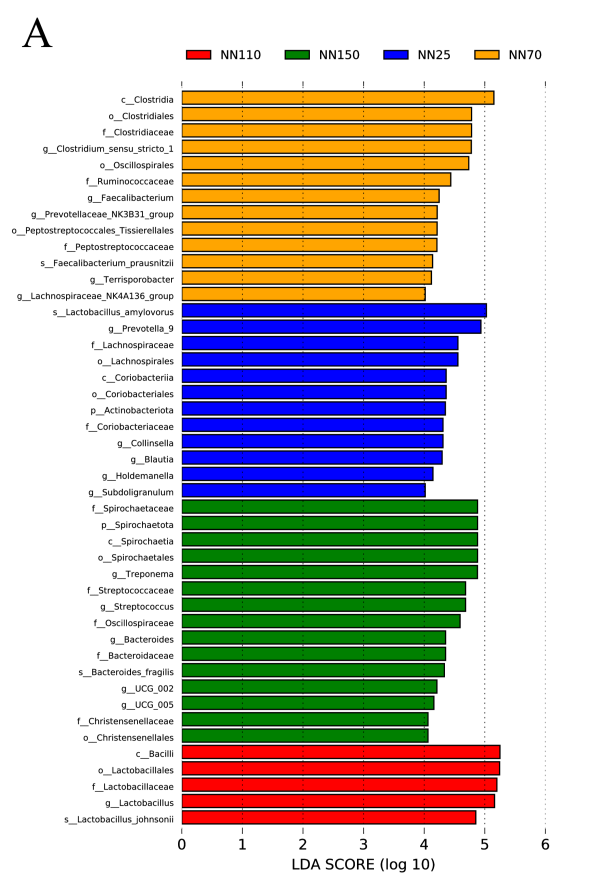

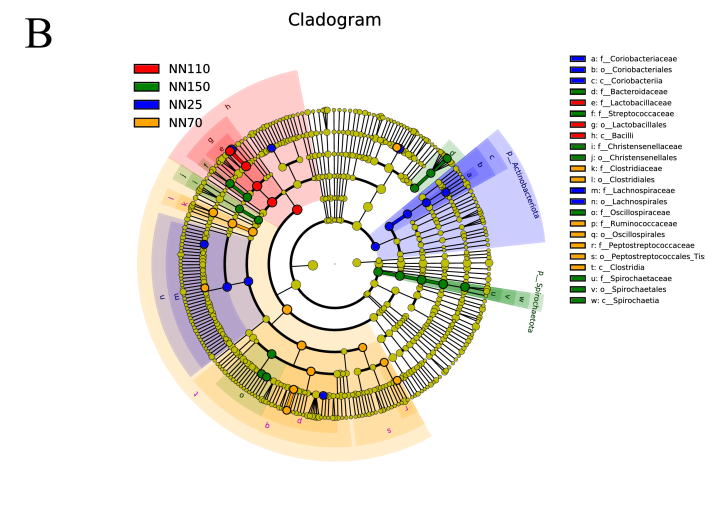


**Figure 5.** (A,B) Differences in the gut microbiota composition of different growth Stages based on Linear discriminant analysis effect size (LEfSe, LDA>4).


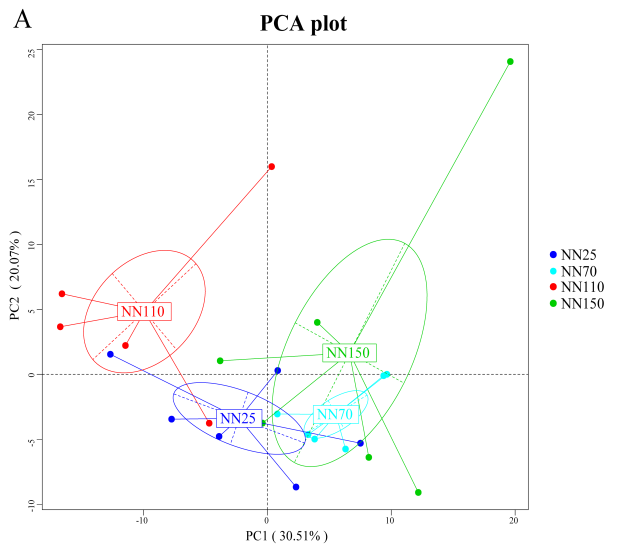

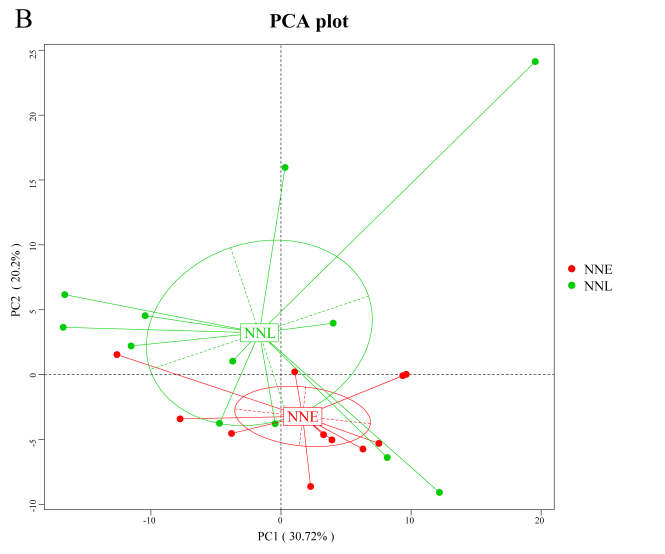


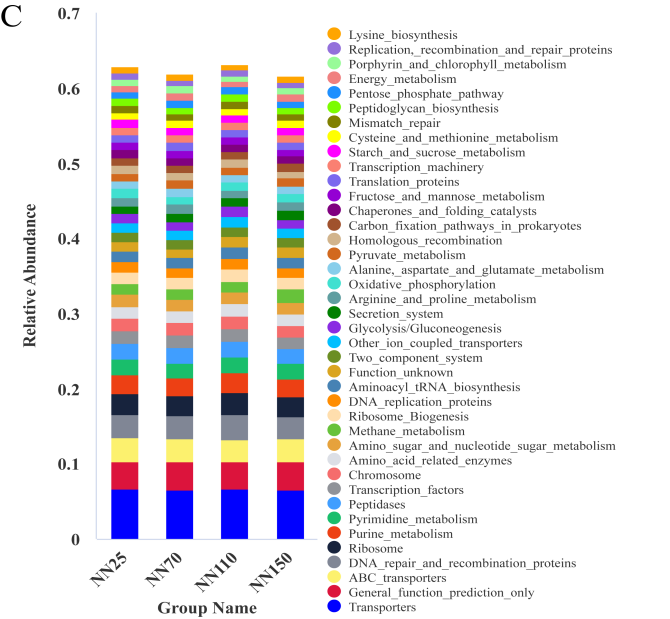

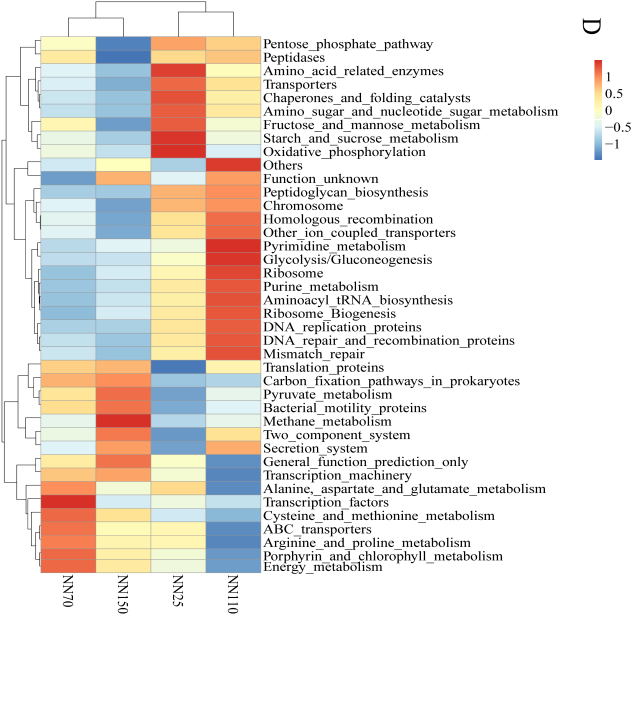


**Figure 6.** KEGG pathway analysis based on PICRUSt2 predicts the functional composition of the gut microbiota in Neijiang pigs at different growth stages. (A, B) Principal component analysis of KEGG pathway abundance. NN25—25 days old; NN70—70 days old; NN110—110 days old; NN150—150 days old; NNE—early growth stage (25 and 70 days old); NNL—finishing growth stage (110 and 150 days old). (C) Histogram of KEGG pathway abundance between different sample groups. (D) Clustering heatmap of KEGG pathway abundance across different sample groups.


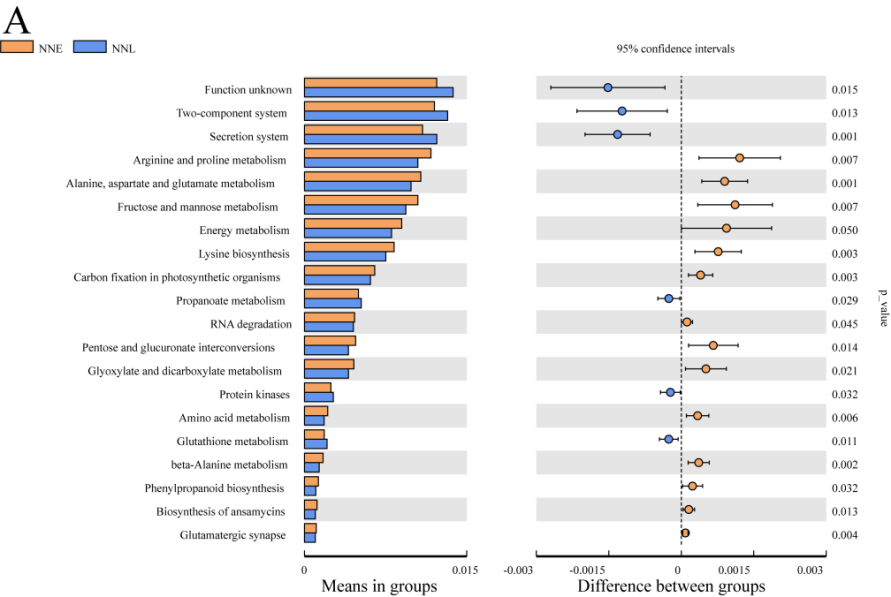

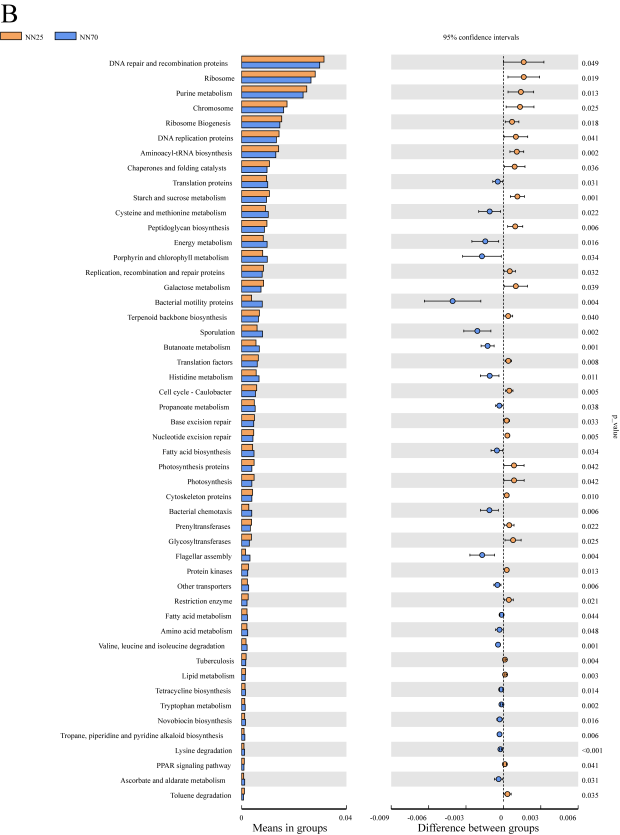


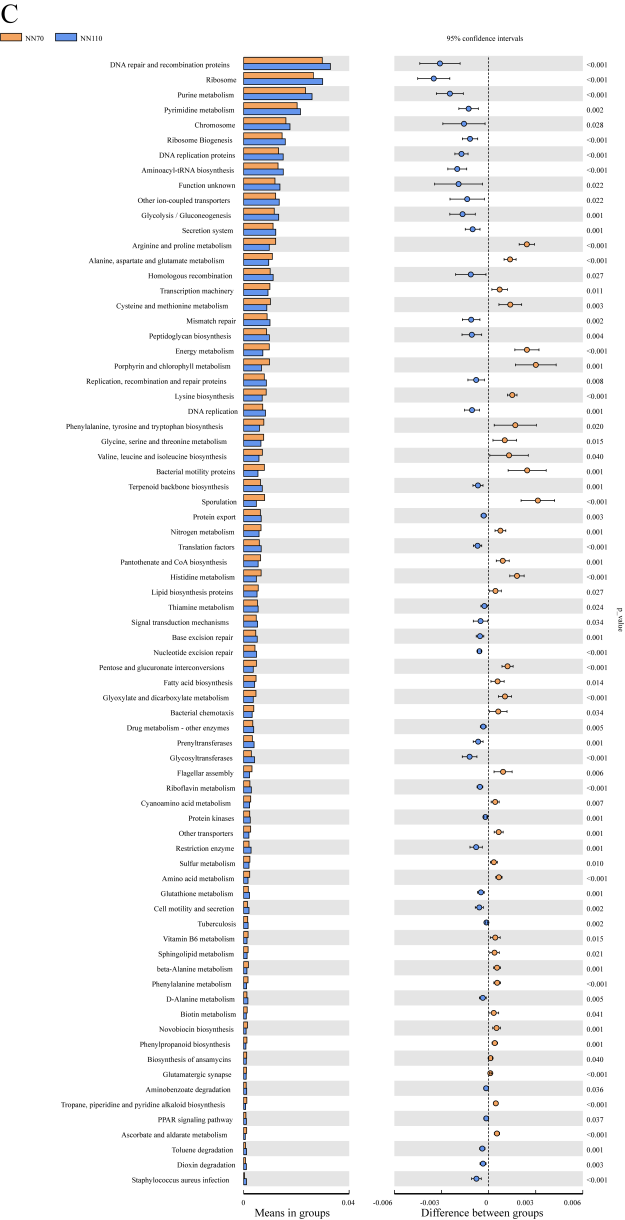

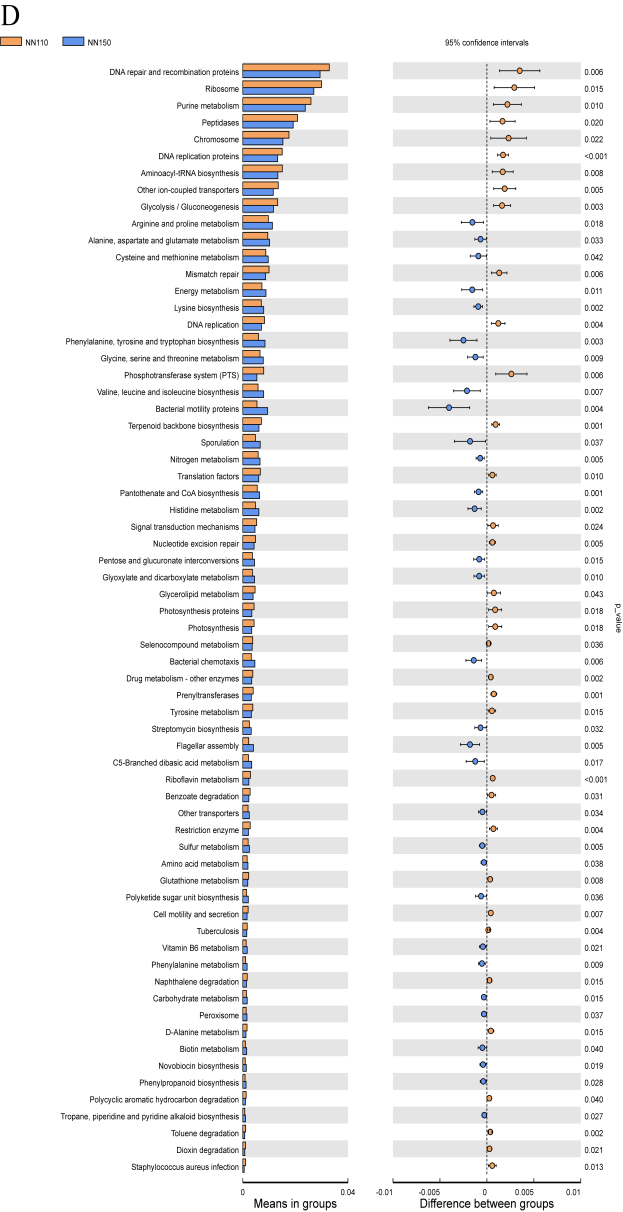


**Figure 7.** Differences in the abundance of annotated KEGG levels (level 3) in the gut microbiota composition of Neijiang pigs at different growth stages based on T-test analysis. (A) NNE vs NNL; (B) NN25 vs NN70; (C) NN70 vs NN110; (D) NN110 vs NN150. NNE—early growth stage (25 and 70 days old); NNL—finishing growth stage (110 and 150 days old); NN25—25 days old; NN70—70 days old; NN110—110 days old; NN150—150 days old. Extended error line plots of the significant differences between the different stages, with corrected p-values shown on the right.
